# Self-Assembly of Modular Bis-MPA Dendrons into Colloidal Particles with Tunable Morphology and Selective Cytotoxicity

**DOI:** 10.3390/nano16070406

**Published:** 2026-03-27

**Authors:** Luis M. Negrón, Clara L. Camacho-Mercado, Cristian A. Morales-Borges, Alondra López-Colón, Ariana De Jesús-Hernández, Ansé E. Santiago-Figueroa, Jean M. Rodríguez-Rivera, Yancy Ferrer-Acosta, Bismark A. Madera-Soto

**Affiliations:** 1 Department of Chemistry, University of Puerto Rico at Cayey, Cayey, PR 00736, USA; cristian.morales5@upr.edu (C.A.M.-B.); alondra.lopez3@upr.edu (A.L.-C.); ariana.de@upr.edu (A.D.J.-H.); anse.santiago@upr.edu (A.E.S.-F.); 2 Department of Biology, University of Puerto Rico at Río Piedras, San Juan, PR 00931, USA; clara.camacho@upr.edu (C.L.C.-M.); bismark.madera@upr.edu (B.A.M.-S.); 3 Department of Natural Sciences, University of Puerto Rico at Cayey, Cayey, PR 00736, USA; jean.rodriguez40@upr.edu; 4 Department of Anatomy & Neurobiology, University of Puerto Rico Medical Sciences Campus, San Juan, PR 00936, USA; yancy.ferrer@upr.edu

**Keywords:** Bis-MPA dendrons, self-assembly, dendritic nanoparticles, colloidal particles, hierarchical morphology, structure–property relationships

## Abstract

Precise control over the physicochemical and biological properties of colloidal particles is essential for the rational design of functional soft materials. In this work, we report a simple and scalable strategy for generating modular dendron particles (MDPs) through the self-assembly of fully characterized small-molecule Bis-MPA dendrons that act as programmable molecular building blocks for colloidal particle formation. By systematically varying three structural domains—the inner functionality, methylene spacer length, and outer connector—we achieve tunable formation of MDPs ranging from nano- to microscale dimensions. Upon solvent evaporation under mild drying conditions, pre-assembled MDPs act as structure-directing seeds that guide the emergence of hierarchical surface morphologies with spiky, scaly, or spherical protrusions, depending on dendron architecture. Importantly, these assemblies exhibit good biocompatibility toward non-tumoral bronchial epithelial (NL-20) cells while displaying selective cytotoxicity toward Neuro-2a neuroblastoma cells, demonstrating that dendron molecular architecture alone can govern particle size, morphology, and biological response without external drug loading. Collectively, these findings highlight modular Bis-MPA dendrons as versatile building blocks for directing particle size, morphology, and biological response through controlled self-assembly and evaporation-driven structuring.

## 1. Introduction

Nanostructured particles with precise control over size, surface morphology, and biocompatibility are central to advances in sensing, catalysis, therapeutics, and biomedical imaging [1,2,3,4,5,6]. Their utility depends heavily on the ability to tailor structural features such as roughness, topology, and shape to influence interfacial interactions, mechanical behavior, and biological outcomes. Although significant progress has been made in synthesizing organic, inorganic, and carbon-based particles with diverse architectures [7,8,9], many reported systems still rely on labor-intensive protocols, limited scalability, or non-biocompatible components [10,11,12]. These limitations hinder their deployment in biological environments, where green, simple, and reproducible fabrication methods are essential.

Irregular or hierarchical morphologies, such as flower-like particles (FLPs), typically require templating agents [6,13,14,15,16,17,18,19] to guide structure formation and morphological growth during processing. However, the removal of such templates can introduce complexity, reduce yield, or compromise biocompatibility. Similarly, oil-in-water emulsion methods often employ solvents such as dichloromethane, which are unsuitable for direct biological use. These challenges highlight the need for a modular, surfactant-free strategy capable of generating particles with tunable surface features through simple self-assembly.

One promising strategy is the structural tuning of small amphiphilic organic molecules that can self-assemble into colloidal structures through noncovalent interactions [20]. Among these, 2,2-bis(hydroxymethyl)propionic acid (Bis-MPA) stands out as a highly versatile building block. Widely used in divergent, convergent, and orthogonal dendrimer synthesis [7,21,22,23,24,25,26,27], Bis-MPA provides a biocompatible, biodegradable polyester scaffold that enables precise control over branching, functionality, and molecular architecture. Importantly, Bis-MPA-based dendrons can be purified and fully characterized at the molecular level prior to assembly, providing a chemically well-defined platform for exploring structure-driven particle formation. Although high-generation Bis-MPA dendrimers have been extensively explored [28,29], the use of simple Bis-MPA dendrons as modular units for particle formation remains underdeveloped. The term “dendron” has been widely used in the literature to describe monodisperse, branched building blocks derived from dendritic architectures, particularly in bis-MPA-based systems. Early studies by Ihre et al. introduced “monodendrons” as well-defined dendritic units, while subsequent work by Malkoch et al. demonstrated the synthesis of bis-MPA-based dendrons via divergent approaches [28,29]. In this work, we use the term “modular dendron” to describe small, simplified dendron-like structures based on bis-MPA building blocks that retain key structural features of dendritic systems, while not corresponding to classical high-generation dendrons.

Here, we introduce a straightforward and scalable strategy for generating modular dendron particles (MDPs) through the self-assembly of fully characterized small-molecule Bis-MPA dendrons that act as programmable molecular building blocks. Because these dendrons contain interchangeable functional domains, they provide an ideal platform for examining how structural variation influences particle size, surface morphology, and biological activity. By systematically modifying the inner functional group (ester or amide), methylene chain length, and connecting moiety (triazole or ester), we demonstrate precise control over the resulting MDP properties. Using common organic transformations such as esterification, S_N_2 substitution, and click chemistry, we constructed a library of five modular dendrons that self-assemble into particles ranging from nano- to microscale dimensions.

This study reveals structure–property relationships that dictate MDP formation, stability, and biological response. We show that small structural changes dramatically alter particle morphology, evaporation-driven dried-state pattern formation, and cytotoxicity profiles. Notably, MDPs exhibit selective cytotoxicity toward Neuro-2a neuroblastoma cells while remaining biocompatible with NL-20 bronchial epithelial cells. Moreover, upon solvent evaporation under mild drying conditions, pre-assembled MDPs act as structure-directing elements capable of generating flower-like or red-blood-cell-like (RBC-like) morphologies—an emergent behavior not previously reported for Bis-MPA dendrons. These observations reveal a processing-driven and morphology-directing mechanism that governs hierarchical structure formation in soft colloidal systems. Collectively, these insights establish modular Bis-MPA dendrons as a versatile platform for bottom-up particle engineering and open up new avenues for designing biomaterials with tunable physicochemical and biological properties.

## 2. Materials and Methods

### 2.1. Materials

All chemicals and solvents were obtained from commercial suppliers and used without further purification unless otherwise noted. Complete experimental procedures, reagent quantities, and characterization data for all compounds are provided in the Supporting Information.

### 2.2. Synthesis of Modular Dendrons (MD 1–5)

Bis-MPA dendron derivatives were synthesized through standard esterification, amide-coupling, and copper-catalyzed azide–alkyne cycloaddition reactions. Detailed synthetic procedures, purification methods, and full spectroscopic characterization for MD 1–5 prior to particle formation are included in the Supporting Information.

### 2.3. Preparation of Modular Dendron Particles (MDPs)

MDPs were generated by dissolving each dendron in aqueous buffer followed by brief ultrasonication to produce uniform colloidal suspensions. No surfactants, templates, or additives were used. Complete preparation protocols are provided in the Supporting Information. All assembly experiments were performed in PBS to mimic biologically relevant conditions. While buffer composition may influence assembly behavior, the observed structures are primarily attributed to intrinsic molecular interactions of the Bis-MPA derivatives. All experiments were conducted at a fixed concentration that consistently yielded stable particle formation. While concentration-dependent effects may influence assembly behavior, a systematic study of these parameters will be explored in future work.

### 2.4. Dynamic Light Scattering (DLS)

Hydrodynamic diameters were measured at 25.0 ± 0.1 °C using standard acquisition parameters. Samples were prepared by sonication of 15 mM dendron solutions in PBS (pH 7.4) for 1 min prior to analysis. Reported hydrodynamic diameters correspond to Z-average values obtained from cumulants analysis of the autocorrelation function. Each sample was measured three consecutive times (*n* = 3, technical replicates). Detailed acquisition settings, data processing procedures, and polydispersity index (PDI) values are provided in the Supporting Information.

### 2.5. Scanning Electron Microscopy (SEM)

Dried MDP samples obtained after solvent evaporation were mounted onto conductive substrates, sputter-coated with a thin metal layer, and imaged under conventional SEM conditions. Instrument settings and specific preparation steps are provided in the Supporting Information. Because particle formation arises from self-assembly of fully characterized Bis-MPA derivatives in aqueous media without added inorganic components, the observed structures are attributed primarily to the dendron building blocks. While techniques such as EDX could provide complementary information regarding potential buffer-associated species, the present study focuses on morphology and structure–property relationships.

### 2.6. Differential Interference Contrast (DIC) Microscopy

DIC images of MDP suspensions were acquired using a Nikon Eclipse Ti-E inverted microscope with A1R confocal system (Nikon Instruments Inc., Melville, NY, USA) operated in transmitted-light mode. Imaging parameters are provided in the Supporting Information.

### 2.7. Cell Culture

Neuro-2a neuroblastoma and NL-20 bronchial epithelial cells were maintained following ATCC guidelines. Media formulations, passage conditions, and handling procedures are described in the Supporting Information.

### 2.8. Cytotoxicity Assay

Cell viability after MDP exposure was quantified using a colorimetric metabolic assay following a 24 h incubation. Concentration ranges, plate layouts, and absorbance-processing methods are included in the Supporting Information.

### 2.9. Statistical Analysis

Statistical methods, sample sizes, and data-analysis procedures are fully described in the Supporting Information.

## 3. Results

### 3.1. Formation and Size Control of Modular Dendron Particles

**Figure 1:**
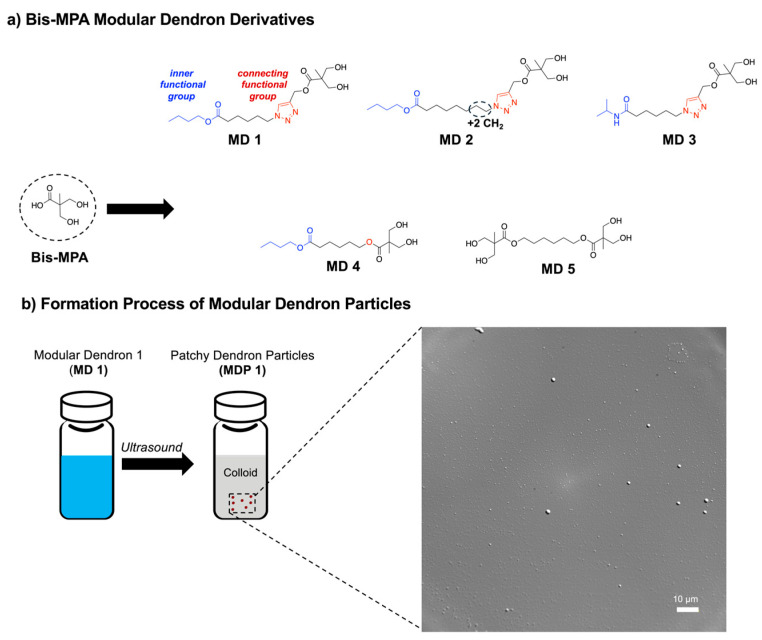
(**a**) Schematic representation of the modular dendron (MD) derivatives synthesized from Bis-MPA, illustrating variations in the inner functional group (blue) and the connecting functional group (red) linking to the terminal Bis-MPA unit. (**b**) Formation process of modular dendron particles (MDPs) from MDs in 1× phosphate-buffered saline (PBS) using ultrasound (40 kHz, 60 W, 1 min). The inset image corresponds to a differential interference contrast (DIC) micrograph of MDPs.

To explore these structure–property relationships, we synthesized the amphiphilic dendron derivative butyl 6-((3-hydroxy-2-(hydroxymethyl)-2-methylpropanoyl)oxy)hexanoate, designated as modular dendron 1 (MD 1) (Figure 1a). All modular dendrons (MDs 1–5) were synthesized as discrete small molecules and purified prior to particle formation; full structural characterization by NMR, IR, and mass spectrometry is provided in the Supporting Information.

**Figure 2:**
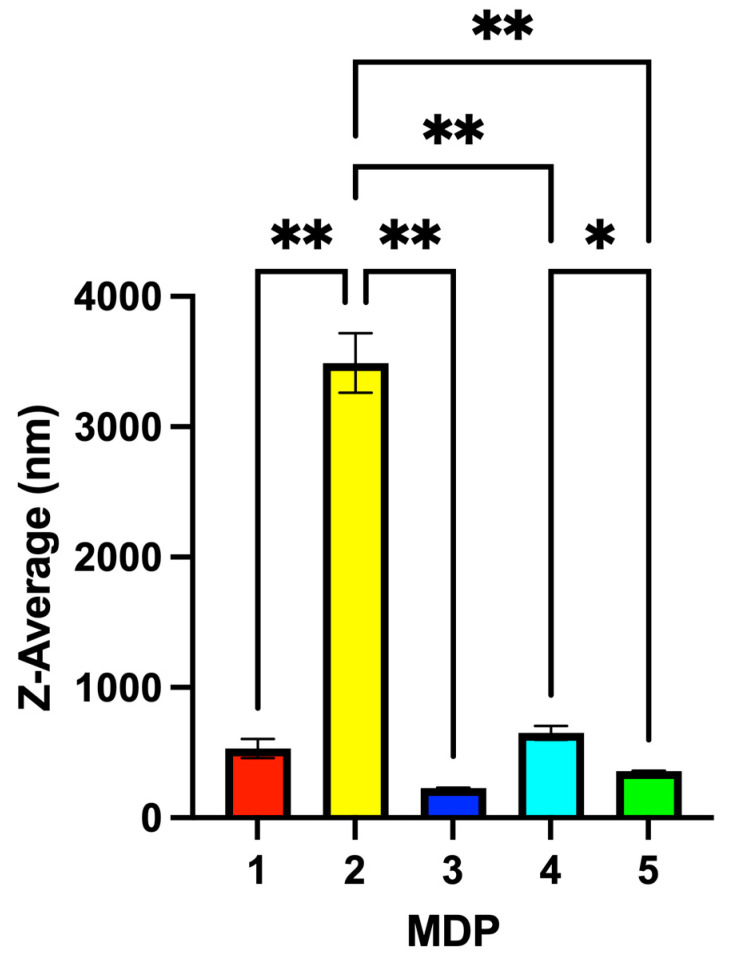
Dynamic light scattering (DLS) analysis showing the Z-average hydrodynamic diameter (Z-Ave, cumulants analysis; mean ± SD, n = 3 technical measurements) of modular dendron particles (MDPs) formed by sonication of 15 mM dendron solutions in PBS (pH 7.4) for 1 min at 25.0 ± 0.1 °C. Detailed DLS acquisition parameters, polydispersity index (PDI) values, and replicate measurements are provided in Table S1 (Supporting Information). Statistical significance: * *p* < 0.05; ** *p* < 0.01.

In this study, the structural components of MD 1 that were varied included the butoxy ester group (serving as the inner functional group) and the triazole linker (the connecting functional group) bridging the inner butoxy segment (bearing five methylene units) to the outer Bis-MPA group. MD 1 exhibits an oily appearance under ambient conditions. Particle formation occurs only upon exposure to aqueous media and ultrasound, indicating that the observed colloidal structures arise from processing-driven self-assembly rather than from pre-existing solid-state organization. This simple protocol for the formation of MDPs does not require external templates or surfactants. These results demonstrate that fully characterized small-molecule Bis-MPA dendrons can act as modular building blocks that self-assemble into colloidal particles without the need for surfactants, templates, or complex fabrication methods. Upon exposure to water and ultrasound, MD 1 self-assembled into a colloidal suspension of particles designated as MDP 1, with an average particle size of 532 nm (Figure 1b and Figure 2).

Based on the structural functionalities of the MD 1 Bis-MPA scaffold, we developed a library of four additional MDs to study their effects on size, dried surface morphology, and cytotoxicity. For MD 2, the derivative was more hydrophobic than MD 1 because the same inner and connecting functional groups were used, but the inner butoxy segment was extended from a five-methylene to a seven-methylene chain. The addition of these two extra methylene units increased the particle size of MDP 2 to 3490 nm (micron scale), more than one order of magnitude larger than MDP 1 (Figure 2). This pronounced size increase likely reflects enhanced hydrophobic interactions arising from the longer alkyl spacer, which promotes the formation of larger colloidal assemblies rather than simple molecular aggregates.

For MD 3, we synthesized an analog of MD 1 in which the butoxy ester was replaced with a N-isopropylamide group attached to a five-methylene chain linked to the Bis-MPA core via a triazole ring. N-Isopropylamide is widely used in materials such as pNIPAM due to its temperature-responsive properties, biocompatibility, and versatility. The resulting MDP 3 displayed an average particle size of 229 nm, very close to that of MDP 1 (Figure 2). The relationship among MDs 1–3 indicates that increasing hydrophobicity through methylene-chain extension has a more dramatic effect on particle size than changing the inner functional group.

After understanding the effects of inner functional group and methylene chain length on particle size, we evaluated the role of the connecting functional group. The triazole moiety is extensively used in dendrimer chemistry due to its rigidity, stability, and directional hydrogen-bonding interactions. As an alternative, dendrons can be connected through esterification. Thus, MD 4 was synthesized by replacing the triazole connector of MD 1 with an ester linkage (Figure 1a). Replacing the triazole with an ester increased the particle size of MDP 4 to 652 nm, almost double the size of MDP 1.

Finally, we prepared a dimeric MD 5 by replacing the butoxy group of MD 4 with another Bis-MPA unit. The resulting MDP 5 displayed a particle size of 359 nm, demonstrating how increased hydrophilicity from the additional Bis-MPA group reduced the overall size (Figure 2). Together, these results highlight how modular chemical design and processing conditions jointly direct particle size across the nano- to microscale. Small structural variations in dendron architecture therefore translate into large differences in particle size and surface organization, revealing a direct structure–property relationship governing MDP formation.

### 3.2. Cytotoxicity and Cell-Type-Dependent Biological Response

To further evaluate how inner and connecting functional groups influence biological response, cell viability studies were conducted using neuroblastoma Neuro-2a (N2a) cells and non-tumoral lung epithelial NL-20 cells. Importantly, all biological assays were performed using freshly prepared colloidal MDP suspensions in PBS rather than dried particles, ensuring that the observed cellular responses reflect interactions with particles in solution. Neuroblastoma represents a widely used *in vitro* model for assessing structure-dependent cytotoxic responses in neuronal cancer cells, providing a relevant platform for evaluating particle–cell interactions [30]. Examination of MDP–cell interactions in both tumoral and non-tumoral models provides insight into how subtle molecular variations modulate biological outcomes.

**Figure 3:**
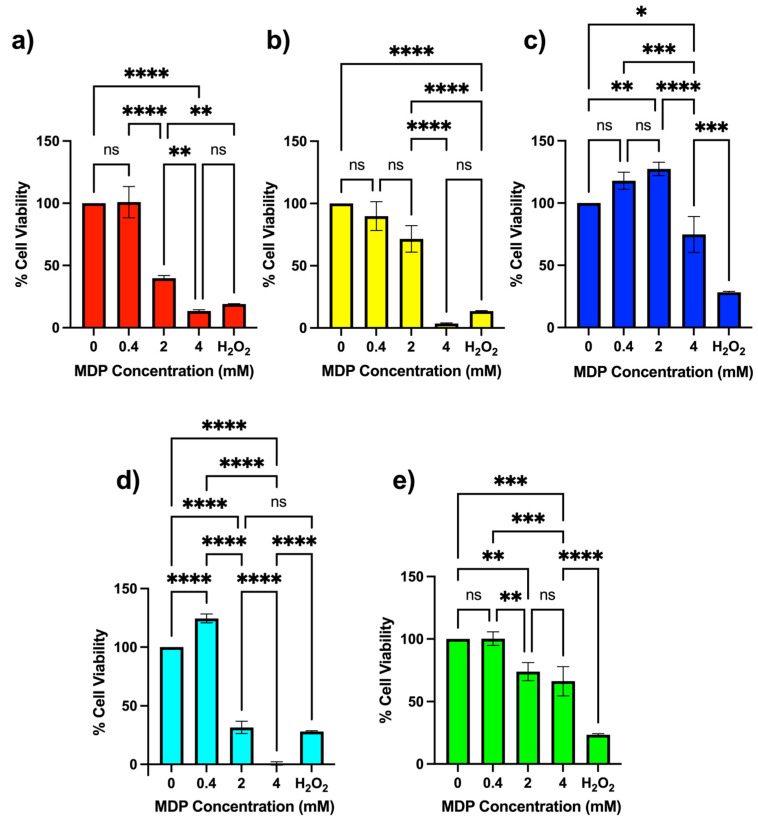
MTS cell-viability assay (mean ± SD, n = 3) for Neuro-2a neuroblastoma cells after 24 h incubation with different concentrations of modular dendron particles (MDPs) prepared from 15 mM dendron solutions in PBS (pH 7.4). Panels correspond to (**a**) MDP 1, (**b**) MDP 2, (**c**) MDP 3, (**d**) MDP 4, and (**e**) MDP 5. Negative values obtained after background subtraction were set to 0% viability because they reflect assay noise rather than biologically meaningful negative cell viability. Statistical significance: * *p* < 0.05; ** *p* < 0.01; *** *p* < 0.001; **** *p* < 0.0001. ns = not statistically significant.

**Figure 4:**
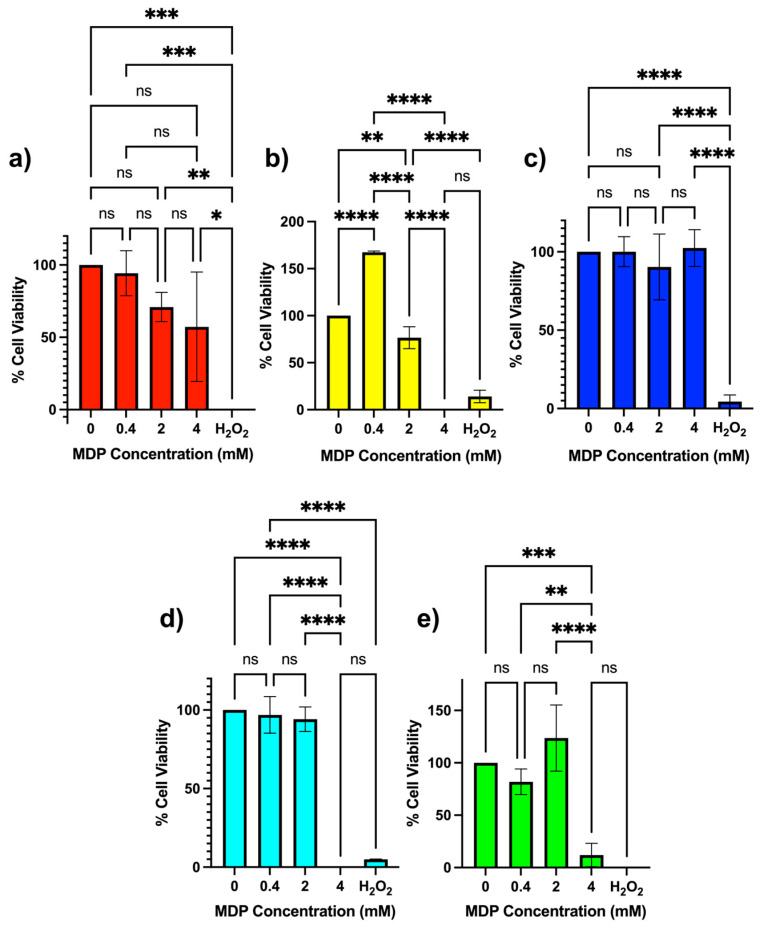
MTS cell-viability assay (mean ± SD, n = 3) for non-tumoral NL-20 bronchial epithelial cells after 24 h incubation with different concentrations of modular dendron particles (MDPs) prepared from 15 mM dendron solutions in PBS (pH 7.4). Panels correspond to (**a**) MDP 1, (**b**) MDP 2, (**c**) MDP 3, (**d**) MDP 4, and (**e**) MDP 5. Negative values obtained after background subtraction were set to 0% viability because they reflect assay noise rather than biologically meaningful negative cell viability. Statistical significance: * *p* < 0.05; ** *p* < 0.01; *** *p* < 0.001; **** *p* < 0.0001. ns = not statistically significant.

According to MTS assays, none of the five MDPs exhibited significant cytotoxicity at 0.4 mM (400 µM) in either cell line (Figure 3 and Figure 4). At 2 mM, however, MDPs 1 and 4 produced marked reductions in N2a viability (<50%), whereas MDPs 2, 3, and 5 maintained viability values above 50% (Figure 3). Notably, MDPs 1 and 4 differ only in their connecting functional group (triazole versus ester), suggesting that small variations in dendron connectivity can significantly influence cellular response.

At 4 mM, this trend became more pronounced. MDPs 1, 2, and 4—each containing a butoxy ester inner functional group—reduced N2a viability to below 13%, whereas MDPs 3 and 5, incorporating an N-isopropylamide or an additional Bis-MPA unit, respectively, retained viability values above 50%. These observations indicate that the nature of the inner functional group plays a dominant role in modulating cytotoxic response in N2a cells.

Importantly, these biological effects arise from particles formed exclusively from small-molecule Bis-MPA dendrons without drug loading or covalent functionalization. Remarkably, these results indicate that dendron molecular architecture alone can induce structure-dependent cellular responses even in the absence of drug loading or covalent functionalization. While most nanoparticle-based systems reported in the literature rely on drug loading or chemical functionalization to induce biological activity [21,31,32,33,34,35], the present results highlight that dendron architecture alone can drive pronounced, structure-dependent cellular responses. Such behavior underscores the utility of modular dendrons as a platform for probing fundamental relationships between molecular design, supramolecular assembly, and biological interaction.

To assess cell-type specificity, analogous MTS assays were performed using non-tumoral NL-20 epithelial cells. At 0.4 mM, all MDPs were non-cytotoxic, displaying viability values near 100% (Figure 4). At 2 mM, MDPs 3–5 maintained full viability, while MDPs 1 and 2 remained above 70%. At 4 mM, MDPs 2, 4, and 5 produced substantial reductions in NL-20 viability, whereas MDP 3 retained nearly complete viability. Although reductions in viability were observed at higher concentrations, the magnitude and pattern of response differed between cell types. Notably, MDP 1 and MDP 4 exhibited differential responses between N2a and NL-20 cells, indicating that specific dendron architectures can elicit cell-type-dependent biological effects. A qualitative summary of these cytotoxicity trends is provided in Table 1.

### 3.3. Evaporation-Driven Morphological Development

**Figure 5:**
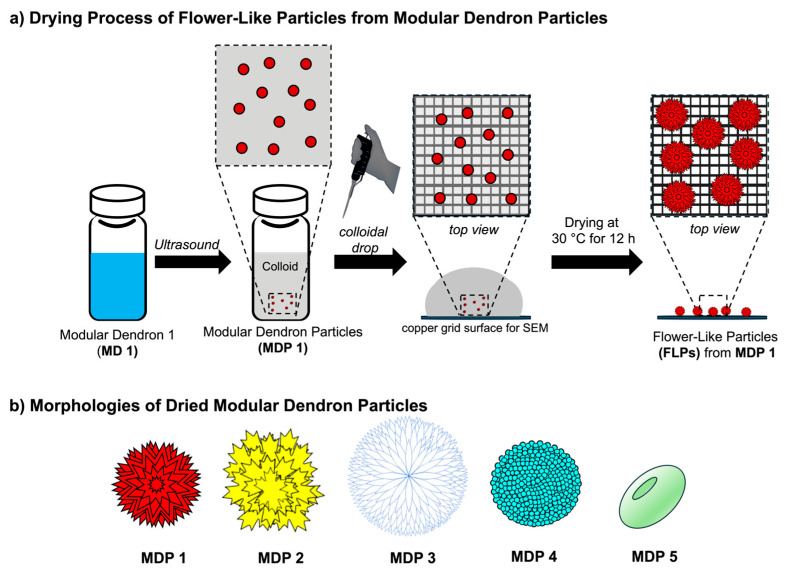
(**a**) SSchematic representation of the drying process in which colloidal modular dendron particles (MDPs) undergo evaporation-driven assembly during mild solvent evaporation, leading to morphology-directing organization. (**b**) Diversity of evaporation-driven surface architectures obtained from different modular dendron particles (MDPs).

**Figure 6:**
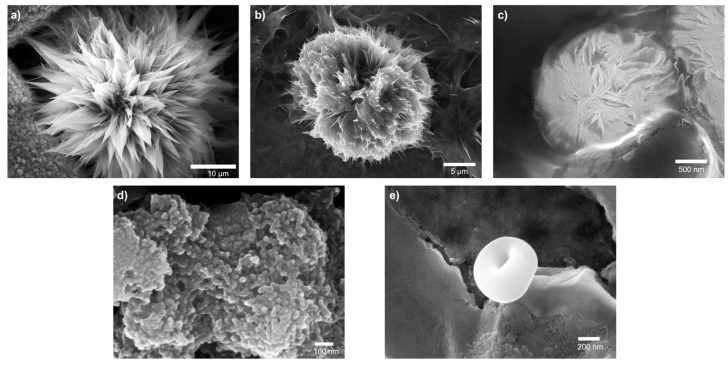
Scanning electron microscopy (SEM) images of dried modular dendron particles (MDPs) formed from 15 mM dendron solutions. (**a**) MDP 1 displaying a spiky flower-like surface architecture; (**b**) MDP 2 exhibiting a scaly surface texture; (**c**) MDP 3 forming a fibrous convex morphology; (**d**) MDP 4 producing uniformly distributed spherical protrusions; and (**e**) MDP 5 adopting a biconcave red-blood-cell-like (RBC-like) morphology. SEM imaging conditions: secondary electron detector (SED), accelerating voltage 10–20 kV, working distance 10–11 mm.

Surface characterization of dried MDPs was conducted using scanning electron microscopy (SEM). A 10 µL aliquot of each colloidal MDP sample was mildly dried at 30 °C for 12 h (Figure 5a). Importantly, this experiment allows for the direct visualization of how pre-assembled colloidal MDPs act as structure-directing seeds during solvent evaporation, providing insight into the processing-driven origin of the final surface morphologies. Remarkably, each MDP developed into a distinct surface morphology during solvent evaporation, indicating a processing-driven, dendron-seeded, morphology-directing assembly process under drying conditions (Figure 5b and Figure 6). The morphologies observed were reproducible across multiple independently prepared samples under identical conditions. SEM revealed that dried MDP 1 formed a spiky flower-like architecture (Dahlia analogy) in the absence of external templating agents. Comparison of the SEM images across the five MDP systems clearly shows that small variations in dendron architecture produce dramatically different surface morphologies, highlighting the strong influence of molecular design on hierarchical particle structure. The diversity of morphologies observed across the MDP series becomes evident when the SEM images are compared side by side (Figure 6). Organic flower-like particles (FLPs) are rare, as most reported FLPs are inorganic [12,36,37,38,39,40], with only a limited number of examples involving hybrid organic–inorganic or polymeric systems prepared using templates. Corrugated surface architectures such as flower-like particles (FLPs), spiky colloids, and other anisotropic morphologies provide enhanced surface area, which is advantageous for catalysis, sensing, and biomedical applications [41,42,43,44]. The observed morphologies are consistent with soft, self-assembled structures rather than crystalline solids. While diffraction-based techniques such as PXRD or electron diffraction could provide additional structural insight, the present study focuses on morphology and structure–property relationships at the mesoscale.

**Figure 7:**
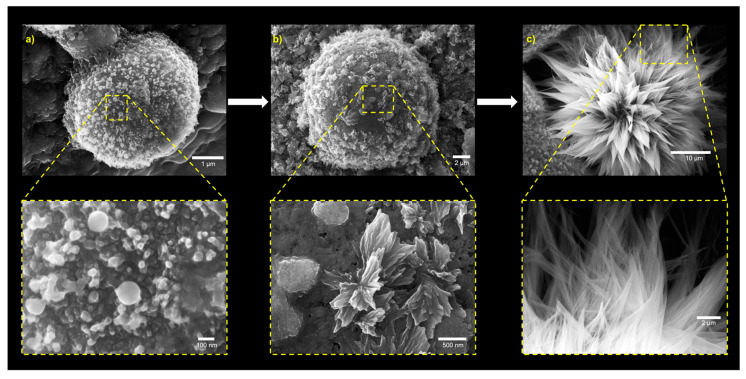
SEM images illustrating the hierarchical, evaporation-driven morphological development leading to the spiky surface architecture of MDP 1 (15 mM MD 1). (**a**) early-stage aggregates; (**b**) intermediate elongation; (**c**) mature spike formation. Lower-magnification images (×2500 and ×5500) reveal early aggregate populations that progressively elongate into protruding surface features, whereas higher magnifications (×7500, ×35,000, and ×100,000) show the formation of mature spike-like structures characteristic of the final flower-like morphology.

A possible origin of the spiky morphology observed for dried MDP 1 is the hierarchical morphological development occurring during solvent evaporation, as evidenced by the presence of multiple populations of intermediate aggregates (Figure 7). Smaller surface features appear to elongate and coalesce into the spike-like protrusions observed in mature structures. Such spiky surfaces may also reduce van der Waals contact between neighboring particles, contributing to improved dispersion stability.

### 3.4. Hierarchical Surface Architectures and Structure–Morphology Relationships

Comparison with other butoxy-containing MDPs (MDPs 2 and 4) revealed pronounced differences in drying-induced surface morphology (Figure 5b, Figures S43 and S45). Dried MDP 2 (Selenicereus undatus analogy) exhibited a scaly surface texture, whereas dried MDP 4 (Craspedia globosa analogy) produced uniformly distributed spherical protrusions rather than elongated features (Figure 6b,d). These observations suggest that the triazole connecting group promotes anisotropic, elongated surface morphology (spiky, flaky, or fibrous), while replacement with an ester linkage (MDP 4) suppresses spike formation.

Dried MDP 3 (Chrysanthemum analogy) displayed a fibrous convex morphology (Figure 6c and Figure S44), consistent with its amide-based connectivity. In contrast, MDP 5, which lacks both the butoxy ester and the triazole connector, adopted a biconcave discoid morphology resembling red blood cells (RBC-like) (Figure 6e and Figure S46). RBC-like architectures are of significant biomedical interest due to their high surface-area-to-volume ratio, deformability, and mechanical resilience [45,46,47,48,49,50,51,52]. Whereas many synthetic RBC mimics require complex fabrication methods (e.g., lithography, seeded polymerization, electrohydrodynamic jetting, or microfluidics), MDP 5 readily adopts an RBC-like morphology under mild drying conditions, highlighting the ability of modular dendron design to direct complex surface architectures through simple processing. Collectively, these results demonstrate that MDP surface morphology can be systematically tuned (from flower-like to RBC-like architectures) by varying key molecular parameters, including the inner functional group, methylene chain length, and connecting moiety. These variations control a drying-driven, morphology-directing assembly process (Table 2). Differences between DLS and SEM sizes are expected due to particle drying and the measurement of hydrodynamic versus dry-state dimensions.

In summary, this study provides fundamental insights into how simple structural modifications within Bis-MPA dendrons govern the self-assembly, size modulation, cytotoxic behavior, and processing-driven morphological organization of pre-assembled soft particles in the resulting MDPs (Table 1 and Table 2). Systematic variation in the inner functional group (ester or amide), hydrophobicity through methylene-chain length, and the presence or absence of the triazole connecting group produced particles ranging from nano- to microscale dimensions without the need for templates, surfactants, or complex fabrication steps. These results establish a scalable and modular strategy for tuning MDP properties through small-molecule design.

Moreover, future studies involving interactions between MDPs and small-molecule drugs or macromolecules (e.g., proteins) may further expand their utility in drug-delivery and biomedical applications. Notably, pronounced, structure-dependent cytotoxic responses in N2a neuroblastoma cells were observed for MDPs 1, 2, and 4, driven by the presence of the butoxy ester inner functionality. In addition, cell-type-dependent cytotoxicity was exhibited by specific MDPs when compared with non-tumoral NL-20 epithelial cells. This behavior is consistent with prior reports showing that hierarchical and flower-like nanoparticle morphologies can exhibit enhanced cytotoxic responses toward cancer cells relative to non-cancerous counterparts [53,54].

Finally, we uncovered a unique processing-driven, morphology-directing behavior in which the combination of the butoxy ester and triazole groups promoted spiky flower-like architectures (MDPs 1 and 2), whereas replacing the triazole with an ester (MDP 4) favored spherical protrusions, and incorporating an amide (MDP 3) yielded fibrous convex morphologies. Notably, MDP 5, containing only a dimeric Bis-MPA unit bridged through a six-methylene linker, produced a red-blood-cell-like structure rather than any flower-like pattern. Collectively, these observations highlight the exceptional versatility of modular Bis-MPA dendrons as programmable building blocks for directing particle size, biological response, and hierarchical morphology through rational molecular design.

## 4. Conclusions

This work demonstrates that simple and modular structural modifications within Bis-MPA dendrons are sufficient to direct the self-assembly of dendron-derived particles (MDPs) with finely tunable physicochemical and biological properties. Systematic variation in the inner functional group, hydrophobic chain length, and connecting moiety yielded self-assembled particles ranging from nano- to microscale dimensions. Importantly, these structures formed in aqueous media without surfactants or complex processing. These modular differences governed particle size and enabled processing-driven, morphology-directing organization during drying, giving rise to hierarchical FLP and RBC-like surface architectures in the absence of external templates.

The observed structure–property relationships were further reflected in biological assays, where MDPs exhibited pronounced, structure-dependent cytotoxic responses in Neuro-2a neuroblastoma cells, while maintaining higher viability in NL-20 bronchial epithelial cells. These results demonstrate that dendron architecture alone can elicit cell-type-dependent biological effects, without the need for drug loading or covalent functionalization.

Overall, this study establishes modular Bis-MPA dendrons as a versatile and scalable design framework for engineering organic particles with programmable size, surface morphology, and biological interactions through simple processing. These findings open up new opportunities for the rational design of soft, dendron-based assemblies for applications in drug delivery, imaging, sensing, and broader materials science contexts.

## Figures and Tables

**Table 1:** Qualitative cytotoxicity trends of modular dendron particles (MDPs) in Neuro-2a and NL-20 cells after 24 h exposure. Concentrations correspond to the MDP concentrations tested in the MTS assays shown in Figure 3 and Figure 4 (0.4, 2, and 4 mM). Viability is expressed qualitatively as +++ (100–80%), ++ (79–50%), + (49–20%), and − (19–0%).

MDP	Neuro-2a Viability (%)			NL-20 Viability (%)		
	0.4 mM	2 mM	4 mM	0.4 mM	2 mM	4 mM
1	+++	+	−	+++	++	++
2	+++	++	−	+++	++	−
3	+++	+++	++	+++	+++	+++
4	+++	+	−	+++	+++	−
5	+++	++	++	+++	+++	−

**Table 2:** Summary of modular dendron particle (MDP) properties as a function of variation in the inner functional group and connecting functional group linking the Bis-MPA outer dendron. Inner and connecting functional groups are highlighted for clarity. For MDPs 1 and 2, the structural difference is the addition of two methylene units in MD 2. * No flower-like pattern observed; RBC-like morphology formed instead.

MDP	Inner Group	Connecting Group	Z-Average Size (nm) ^1^	Surface Morphology
1	Ester	Triazole	532	Spike
2	Ester	Triazole	3490	Flakes
3	Amide	Triazole	229	Fibers
4	Ester	Ester	652	Spherical protrusions
5	Ester	Bis-MPA dimer	359	Biconcave *

^1^ Particle sizes correspond to Z-average hydrodynamic diameters obtained from cumulants analysis of DLS measurements (see Table S1 for mean ± SD values).

## Data Availability

The data supporting the findings of this study are available within the article and its Supplementary Materials.

## Supplementary Materials

The following supporting information can be downloaded at: https://www.mdpi.com/article/10.3390/nano16070406/s1. The Supporting Information includes detailed experimental procedures for the synthesis, purification, and structural characterization of all modular dendrons (MD 1–5); NMR, FTIR, and ESI-MS spectra; protocols for dynamic light scattering (DLS), scanning electron microscopy (SEM), and MTS cell-viability assays; differential interference contrast (DIC) microscopy details; procedures for MDP formation; and extended cell-culture and data-analysis methods, and additional figures (Figures S1–S46) and tables (Table S1). References [55,56] are cited in the supplementary materials.

## Abbreviations

The following abbreviations are used in this manuscript:

Bis-MPA	2,2-bis(hydroxymethyl)propionic acid
MD	modular dendron
MDP	modular dendron particle
FLP	flower-like particle
RBC-like	red blood cell-like
PBS	phosphate-buffered saline
DLS	dynamic light scattering
SEM	scanning electron microscopy
DIC	differential interference contrast
MTS	3-(4,5-dimethylthiazol-2-yl)-5-(3-carboxymethoxyphenyl)-2-(4-sulfophenyl)-2H-tetrazolium assay
