# Supplementary material for: Self-Assembly of Modular Bis-MPA Dendrons into Colloidal Particles with Tunable Morphology and Selective Cytotoxicity
Source: Nanomaterials (Basel). 2026 Mar 27;16(7):406. doi: 10.3390/nano16070406 (PMC13075144; doi:10.3390/nano16070406)
Supplement: Supplementary file 1 [file nanomaterials-16-00406-s001.zip › nanomaterials-4224626-supplementary.pdf]

Self-Assembly of Modular Bis-MPA Dendrons into Colloidal Particles with Tunable Morphology and Selective Cytotoxicity

Authors:
Luis M. Negrón*[1], Clara L. Camacho-Mercado[2], Cristian A. Morales-Borges[1], Alondra López-Colón[1], Ariana De Jesús-Hernández[1], Ansé E. Santiago Figueroa[1], Jean M. Rodríguez-Rivera[3], Yancy Ferrer-Acosta[4], and Bismark A. Madera-Soto[2]

Affiliations:
[1] Department of Chemistry, University of Puerto Rico at Cayey, Cayey, PR 00736
[2] Department of Biology, University of Puerto Rico at Río Piedras, San Juan, PR 00931
[3] Department of Natural Sciences, University of Puerto Rico at Cayey, Cayey, PR 00736
[4] Department of Anatomy & Neurobiology, School of Medicine, University of Puerto Rico Medical Sciences Campus, San Juan, PR 00936

Corresponding author: *luis.negron11@upr.edu*

Table of Contents

I. General experimental procedures........ 2

II. Instrumentation used for characterization........ 2

III. Synthesis and characterization........ 3

IV. MDP Formation Protocol 26

V. Scanning Electron Microscopy (SEM) characterization........ 27

VI. Cell culture studies........ 29

VII. MTS cell viability studies........ 29

VIII. Dynamic Light Scattering (DLS)........ 30

IX. References........ 31

I. General experimental procedures

All reagents and solvents were purchased from commercial sources and used without further purification or treatment. For the synthesis and purification of **Modular Dendrons (MD) 1-5** (Figure S1), the solvents tetrahydrofuran (THF), methylene chloride, toluene, acetone, n-hexane, ethyl acetate, 1X phosphate buffered saline (PBS), and methanol were purchased from Fisher Scientific. Other materials purchased from Fisher Scientific includes magnesium sulfate, potassium sodium carbonate, sodium azide, and potassium iodide. In the case of organic reagents 6-bromohexanoic acid, 8-bromooctanoic acid, L-ascorbic acid, and 1,6 hexanediol were purchased from Sigma Aldrich. Materials such as 4-Dimethylaminopyridine (DMAP), p-toluenesulfonic acid (PTSA), DOWEX 50WX2 resin, $CDCl_3$ and DMSO-d_6 were purchased from Acros Organics. Other materials used like 2,2-bis(hidroxymethyl)-propionic acid, n-butanol, 2-2-dimethoxypropane, and silica gel 60 (L14003) were purchased from Alfa Aesar. Compound purification was performed by column chromatography using silica gel 60 (0.04-0.063 mm) and followed by thin layer chromatography (TLC) using EMD silica gel 60 F254 glass backed plates (particle size 10 to 12 µm, pore size 60 Å) from MilliporeSigma. Unless stated, all the reported retention factors (Rf) values were obtained by TLC visualization with iodine stain.

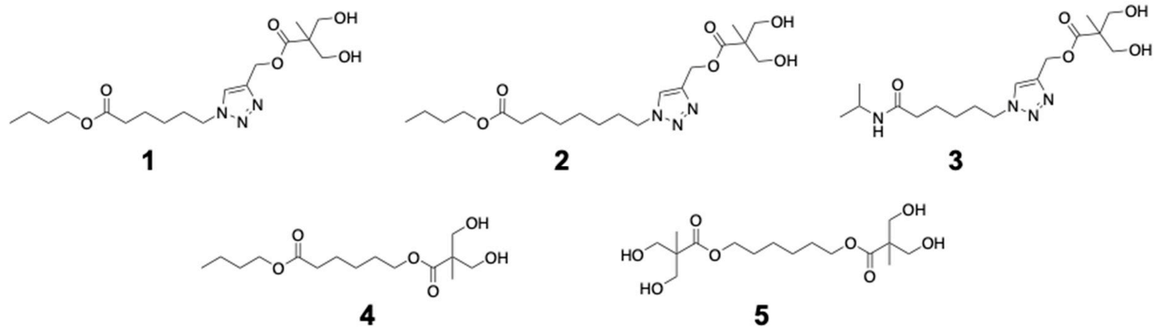

Figure S1. Synthesized Bis-MPA Modular Dendrons (MD) used for the formation of Modular Dendron Particles (MDPs).

II. Instrumentation used for characterization

The ^{1}H NMR characterization was performed on Ascend Aeon 500 MHz NMR from Bruker recorded in the software TopSpin Version 3.5 (patch level 7). The ^{1}H NMR data was processed by using the software MestReNova 14.3.3. For all the characterized compounds in ^{1}H NMR, the samples were prepared on a concentration of 8 mg/mL using DMSO-d_6 (except compound **D** that was dissolved in $CDCl_3$). Also, Fourier Transform Infrared Spectroscopy (FTIR) was performed for compound characterization by using a Nicolet Si10 (Software: OMNIC 9, version 9.8) from Thermo Fisher Scientific with Smart iTR ATR Diamond accessory. For final compounds **1-5**, Electrospray ionization mass spectrometry (ESI-MS) was performed with a Xevo G2-S QTof from Waters. The setup was in positive polarity, 30 V (cone voltage), 6 V (collision energy), flow rate of 30.00 µL/min, and the software used to process the data was MassLynx 4.1 (SCN884). Samples were dissolved in methanol HPLC grade with 1 % formic acid, each sample concentration for the ESI-MS study was 10 ppm.

Dynamic light scattering (DLS) measurements were performed at 25.0 ± 0.1 °C using a Zetasizer Nano ZS (model ZEN3600, Malvern Instruments Ltd.). Hydrodynamic diameter (D_H) values

were obtained in triplicate, with each measurement consisting of an average of 11 runs following an equilibration time of 60 s. Data were analyzed using the cumulants method implemented in the instrument software (Malvern Zetasizer Software v7.10). The dispersant was PBS (0.01 M, pH 7.4), with a refractive index of 1.330 and a viscosity of 0.8882 mPa·s. Measurements were performed using a 4 mW He–Ne laser (λ = 632.8 nm), a backscattering angle of 173°, measurement position of 4.64 mm, and attenuator setting of 11. Samples consisted of 15 mM solutions of modular dendrons (**MDP 1–5**) in PBS (1×).

For the microscopy experiments, a JEOL JSM-IT500HR/LA scanning electron microscope (SEM) coupled with a DRY SD30 energy-dispersive spectroscopy (EDS) detector was used. Instrument control and image processing were carried out using JEOL SEM and SMILEVIEW Lab software. Samples were gold-coated on holey carbon-supported copper grids (200 mesh, 100 nm) using a Pelco SC-7 Auto Sputter Coater with a gold target.
Differential interference contrast (DIC) microscopy was performed using a Nikon Eclipse Ti-E inverted microscope equipped with a Nikon A1 imaging system operated in transmitted-light mode. DIC images were processed using NIS-Elements software (version 6.0). MDP samples were placed in 2-well glass-bottom chamber slides at a concentration of 15 mM of modular dendrons (MDPs 1–5) in 1× PBS.

III. Synthesis and characterization

A. Procedure for the synthesis of prop-2-yn-1-yl 3-hydroxy-2-(hydroxymethyl)-2-methylpropanoate (compound *D*)

For the synthesis of Modular Dendrons, the terminal dendron **B**, **C** and **D** were prepared by following previous reported procedures from Hult[1,2] and coworkers (Figure S2). The first intermediate 2,2,5-trimethyl-1,3-dioxane-5-carboxylic acid (**B**) was prepared by adding the **starting material A** (10.0 g, 74.6 mmol), PTSA (0.71 g, 4.1 mmol) and 25 mL of acetone in a dried round bottom flask. Once the starting material was partially soluble, 2,2-dimethoxy propane (14 mL, 114 mmol) was added to the reaction with a stirring time of 2h. After 2h, 0.3 mL of a 1:1 ethyl acetate/NH_3 was added to the reaction mixture. The acetone was removed by rotary evaporation at 31°C until a white solid was formed. The white solid was dissolved in 250 mL of ethyl acetate and transferred to a separation funnel to be washed with three portions of 20 mL of distilled water. The organic phase was dried with $MgSO_4$ anhydrous, and vacuum filtered to remove the drying agent. Finally, the ethyl acetate from the organic phase was removed by rotary evaporation at 31°C and the final product was kept in vacuum overnight. The final product **B** was then collected in the round bottom flask as a white solid (54 % yield) and monitored by ^{1}H NMR (Figure S3).

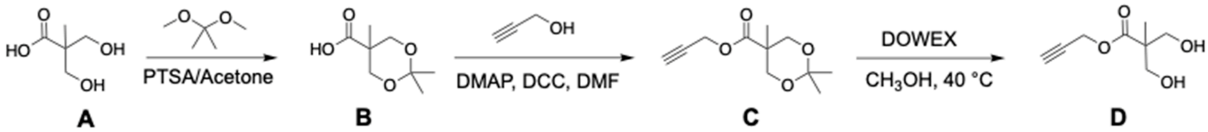

Figure S2. Synthetic scheme for terminal dendrons of 2,2,5-trimethyl-1,3-dioxane-5-carboxylic acid (**B**) and prop-2-yn-1-yl 3-hydroxy-2-(hydroxymethyl)-2-methylpropanoate (**D**) from 2,2-bis(hydroxymethyl)propionic acid (**A**).

A Steglich esterification was performed for the preparation of Intermediate prop-2-yn-1-yl 2,2,5-trimethyl-1,3-dioxane-5-carboxylate **(C)** by adding **B** (1.5 g, 8.6 mmol), DMAP (52 mg, 4.3 mmol), propargyl alcohol (1.5 mL, 25.8 mmol)**,** and 25 mL of CH_2Cl_2 in a dried round bottom

flask. Once the reaction mixture was partially dissolved, N, N- dicyclohexylcarbodiimide (DCM) (2.7 g, 13.0 mmol) was added for a reaction time of 12 h. Product **C** formation was monitored by TLC (R_f = 0.80, 7:3 hexane/ethyl acetate) and ^{1}H NMR (Figure S4). The reaction mixture was vacuum filtered to remove the white solid dicyclohexyl urea, followed by column chromatography using a solvent gradient from 95:5 to 75:25 of hexane/ethyl acetate to obtain an oily product **C** (81.7 % yield).

Finally, prop-2-yn-1-yl 3-hydroxy-2-(hydroxymethyl)-2-methylpropanoate (**D**) was obtained from the deprotection of intermediate **C** with DOWEX 50WX2 resin. In a round bottom flask, compound **C** (1.8 g, 8.5 mmol) was dissolved in 20 mL of methanol. Followed by the addition of 1.8 g of DOWEX 50WX2 resin to let the reaction mixture for 12 h at 40 °C. The reaction was monitored by TLC (R_f = 0.15, 7:3 hexane/ethyl acetate), once the product was formed the solid DOWEX 50WX2 resin was vacuum filtered and the methanol was removed by rotary evaporation to obtain an amber color oil product **D** (78.9 % yield) observed by ^{1}H NMR (Figure S5).

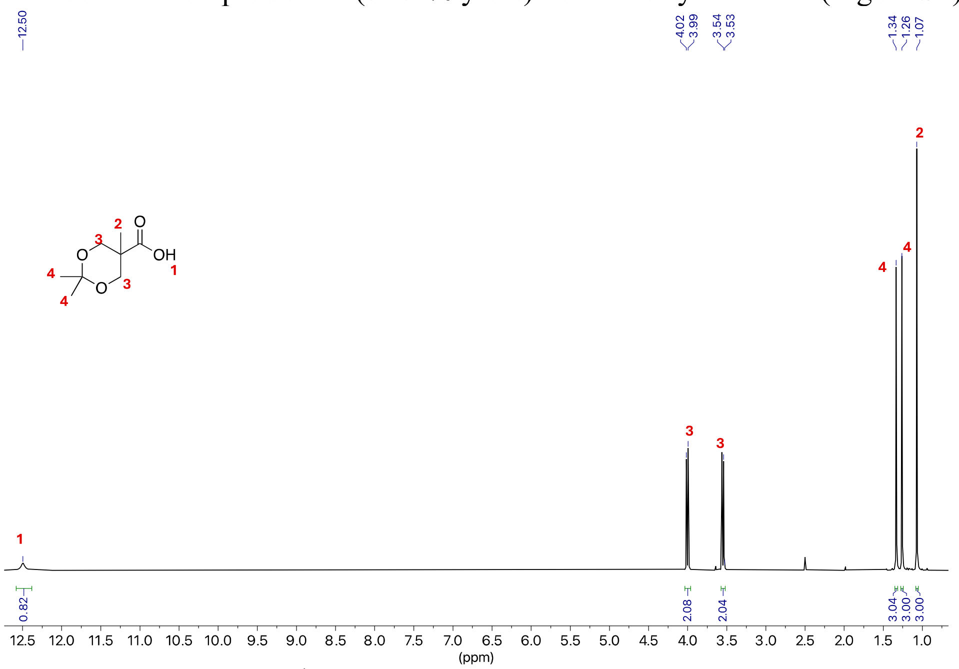

Figure S3. ^{1}H NMR (DMSO–d_6, 500 MHz, 298 K) of **B**.

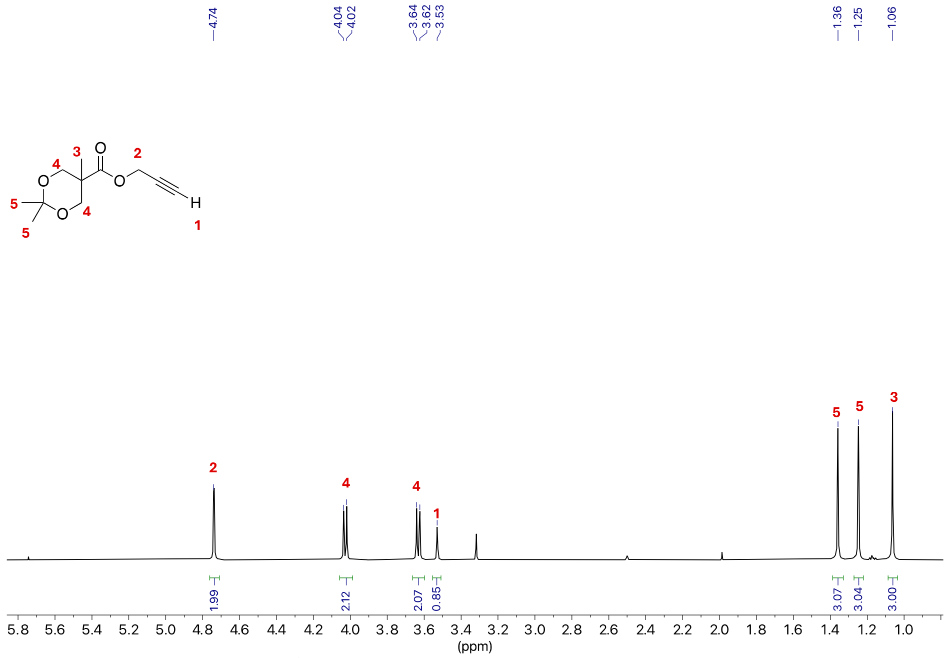

Figure S4. ^{1}H NMR (DMSO–d_6, 500 MHz, 298 K) of **C**.

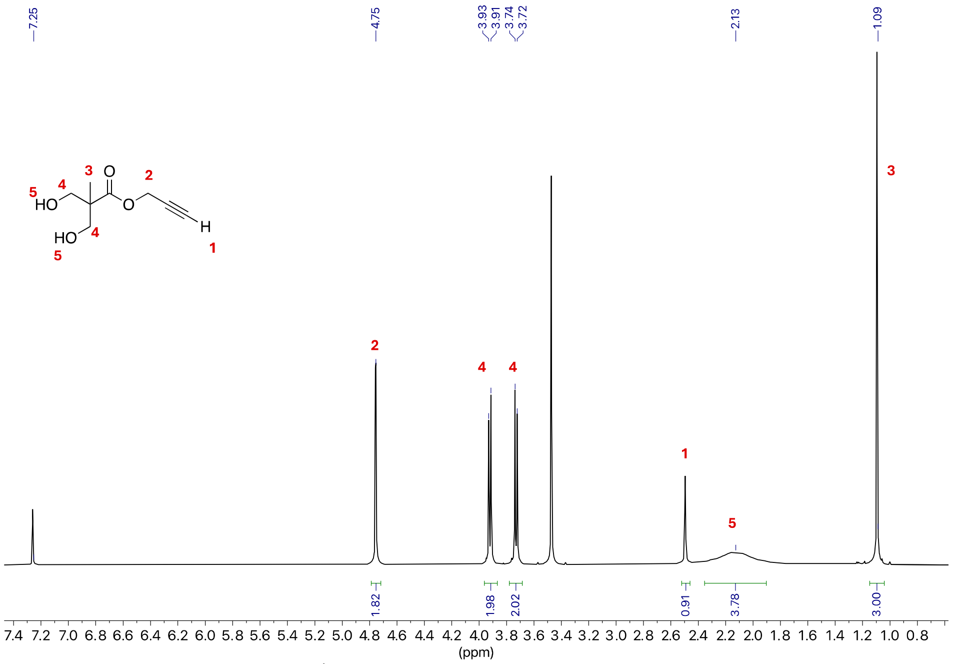

Figure S5. ^{1}H NMR ($CDCl_3$, 500 MHz, 298 K) of **D**.

B. Synthesis and characterization of compounds 1 and 2

Compounds butyl 6-((3-hydroxy-2-(hydroxymethyl)-2 methylpropanoyl)oxy) hexanoate (**1**) and butyl 8-(4-(((3-hydroxy-2-(hydroxymethyl)-2- methyl propanoyl) oxy) methyl)-1H-1,2,3-triazol-1-yl)octanoate (**2**) were synthesized from 6-bromohexanoic acid (**1a**) and 8-bromooctanoic acid

(**2a**) respectively (Figure S6). The following procedure explained for compound **1** was the same procedure used for compound **2**.

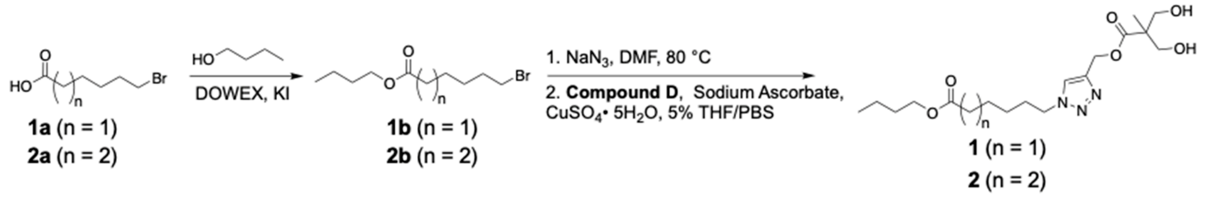

Figure S6. Synthetic scheme of Modular Dendrons **1** (from 6-bromohexanoic acid **1a**) and **2** (from 8-bromooctanoic acid **2a**).

Esterification of **1a** (and for 2a) was performed by using a methodology reported by Turhanen.[3] First, 5g of DOWEX 50WX2 resin was mixed with 10 mL of 2 M HCl solution, followed by a stirring at room temperature (25 °C) for 30 min. After 30 min, the DOWEX 50WX2 resin was vacuum filtered and washed with deionized water until the pH of the filtrated solution was neutral (pH 7.0). The DOWEX 50WX2 resin was dried in an oven for 18 h at 120 °C.

Once the treated DOWEX 50WX2 resin was dried, the reaction to obtain **1b** was performed by mixing in a round bottom flask 0.15 g DOWEX 50WX2 resin with **1a** (1.5 g, 7.7 mmol), KI (42 mg, 0.25 mmol), 1-butanol (5.3 mL, 57.9 mmol). The mixture was stirring under reflux at 65 °C for 4 h and product **1b** was monitored by TLC (R_f = 0.90 (**1b**), 0.87 (**2b**), 100 % ethyl acetate). After reaction completion, the DOWEX 50WX2 resin was removed by vacuum filtration, followed by rotary evaporation of butanol until a dark brown reddish oil was obtained. The crude oil was dissolved in 25 mL of CH_2Cl_2 and transferred to a separation funnel. The CH_2Cl_2 was washed with two portions of 5 mL 10 % $Na_2S_2O_3$ solution. Then, the extracted CH_2Cl_2 phase was dried with $MgSO_4$ anhydrous (drying agent), followed by vacuum filtration to remove the drying agent to finally remove the filtrated CH_2Cl_2 by rotary evaporation. Compound **1b** (50.7 % yield) and **2b** (90.7 % yield) were identified and characterized by ^{1}H NMR and FT IR (Figures S7-S10).

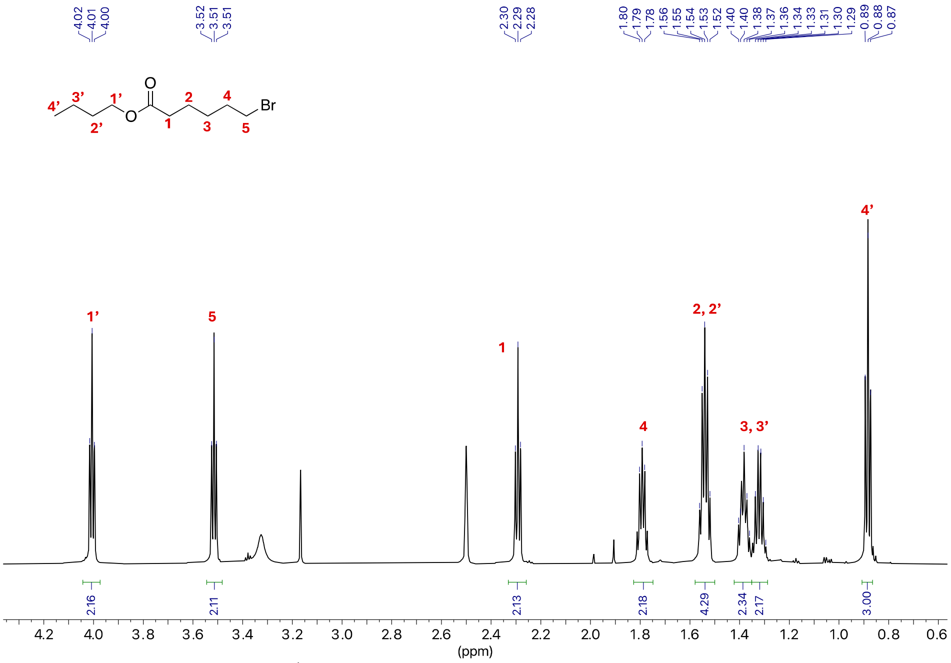

Figure S7. ^{1}H NMR (DMSO–d_6, 500 MHz, 298 K) of **1b**.

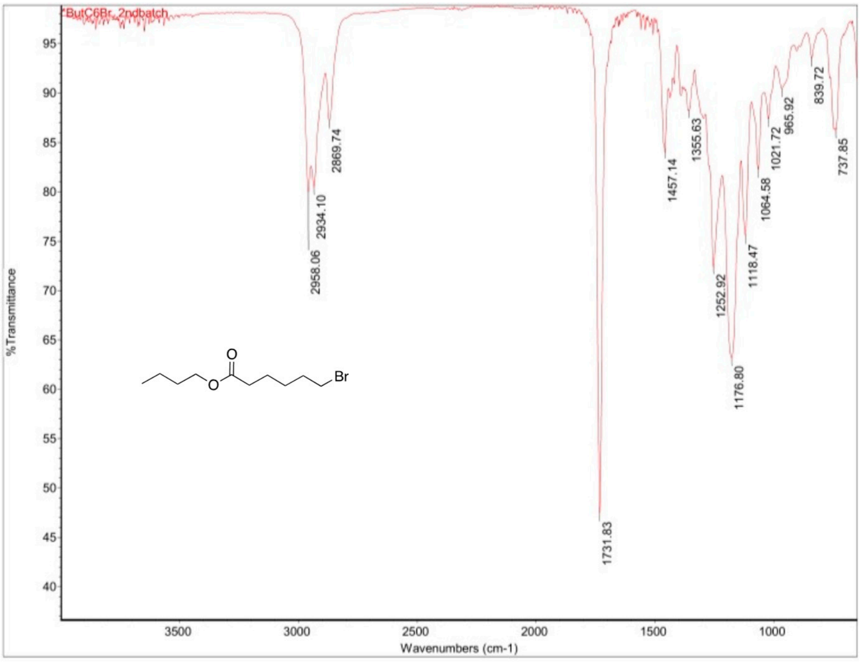

Figure S8. FTIR spectrum of intermediate **1b** at 298 K.

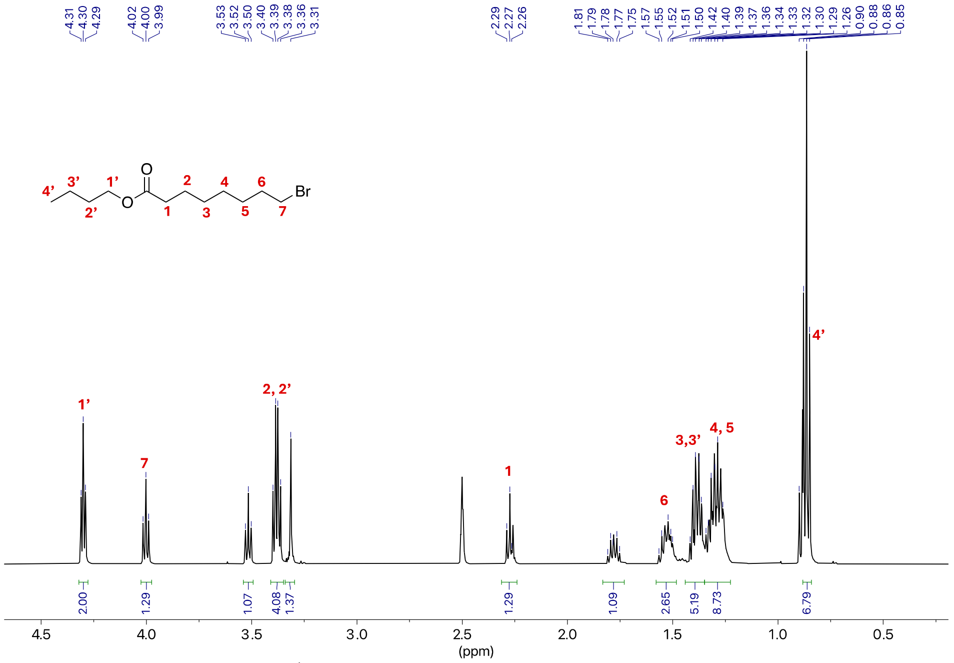

Figure S9. ^{1}H NMR (DMSO–d_6, 500 MHz, 298 K) of **2b**.

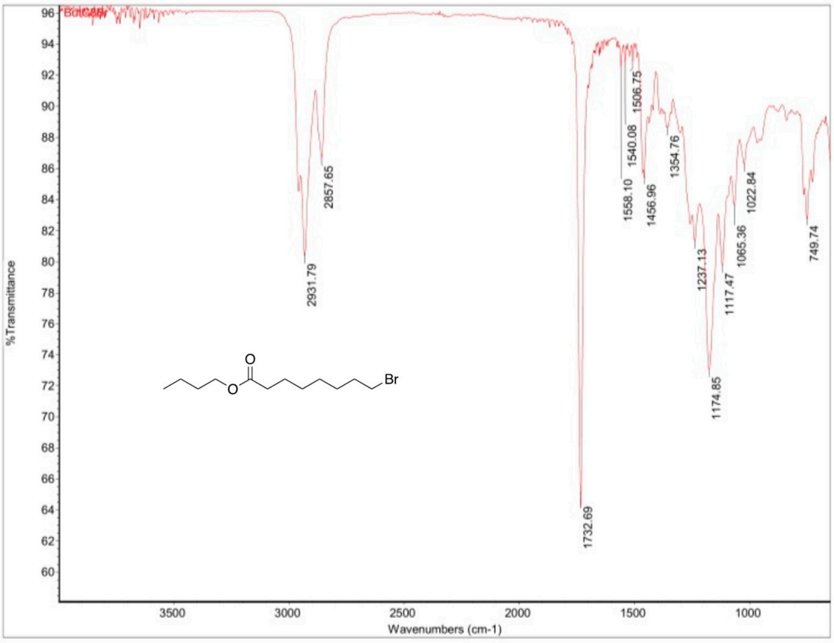

Figure S10. FTIR spectrum of intermediate **2b** at 298 K.

Like the previous described procedure for **1b** and **2b**, compounds **1** and **2** were synthesized using the same following procedure. For the preparation of **1**, **1b** (760 mg, 3 mmol) was mixed with NaN_3 (787 mg, 12.1 mmol) in a round bottom flask with 10 mL of DMF for a reaction of 12 h at 80 °C. The DMF was removed by rotary evaporation, by adding 10 mL of toluene to the reaction mixture until a solid crude was formed. The solid crude was dissolved in 25 mL of methanol and the precipitated solid NaN_3 was removed by vacuum filtration. The methanol was removed by

rotary evaporation and an oily reddish product **1c** (R_f = 0.96 (**1c**), 0.91 (**2c**), 100% ethyl acetate) was obtained in the round bottom flask that was used to perform the click reaction with compound **D**. Products **1c** (64.5 % yield) and **2c** (60 % yield) were characterized by 1H NMR and FT IR (Figures S11-S14).

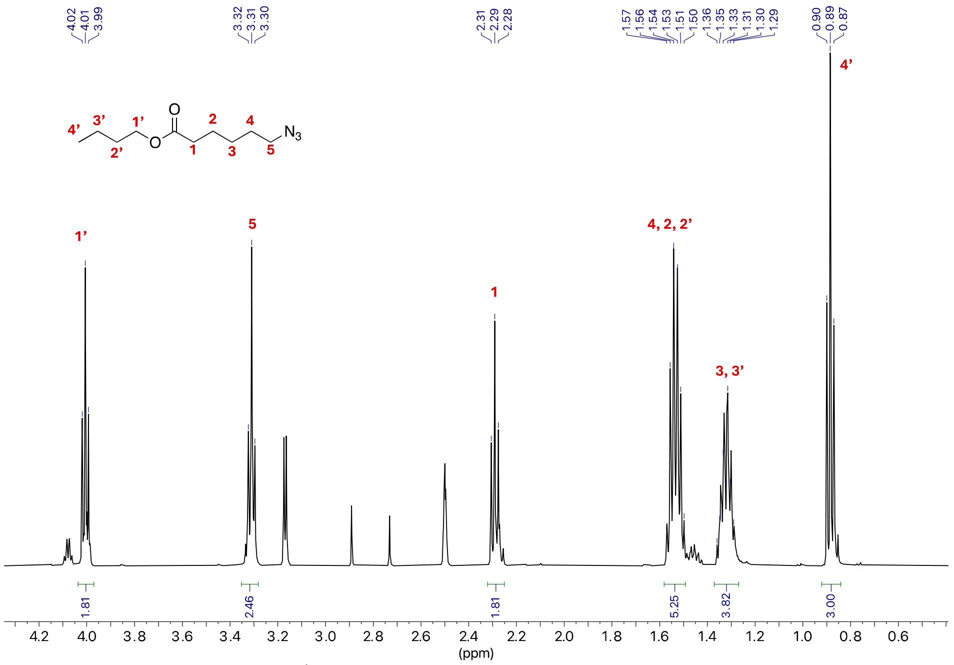

Figure S11. ^{1}H NMR (DMSO–d_6, 500 MHz, 298 K) of **1c**.

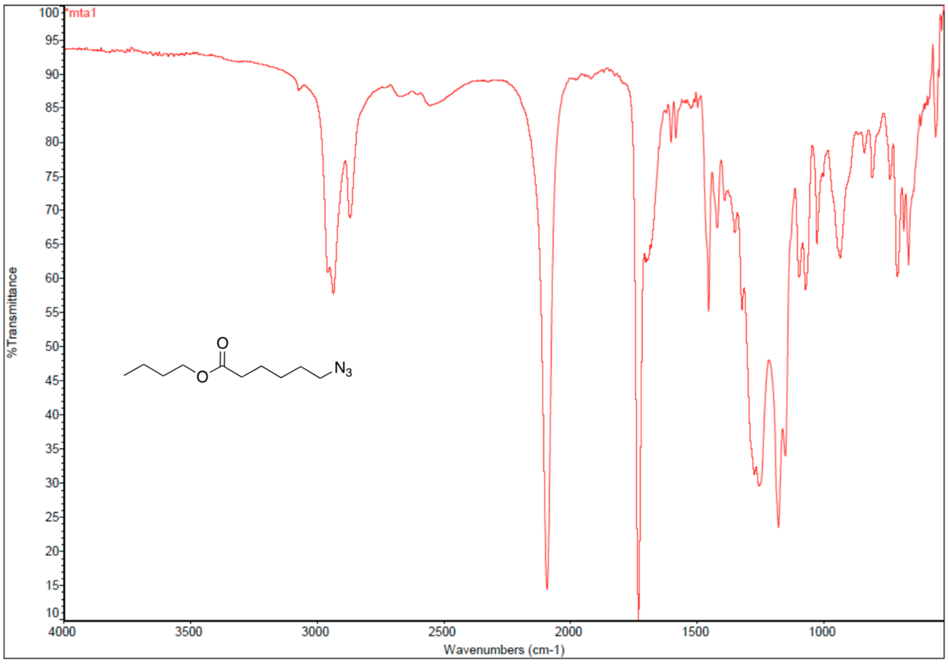

Figure S12. FTIR spectrum of intermediate **1c** at 298 K.

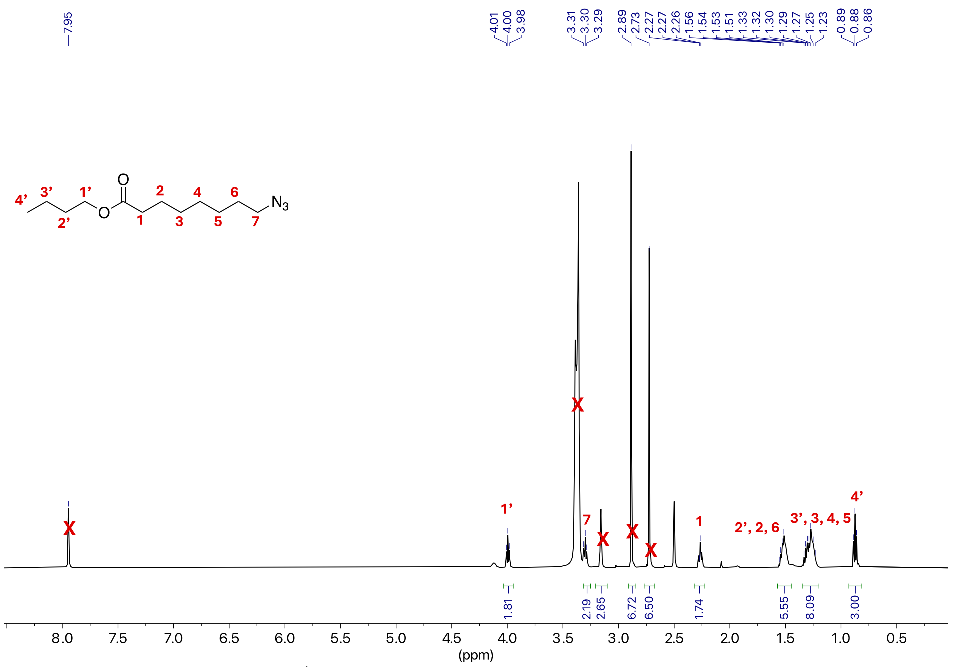

Figure S13. ^{1}H NMR (DMSO–d_6, 500 MHz, 298 K) of **2c**.

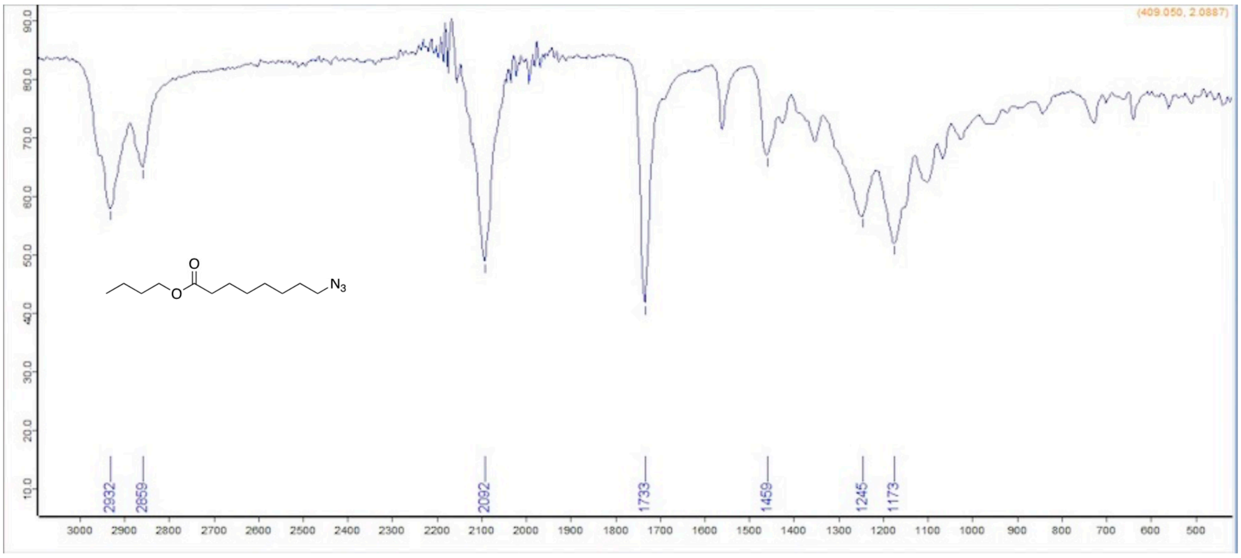

Figure S14. FTIR spectrum of intermediate **2c** at 298 K.

For the click reaction to obtain compound **1**, in a round bottom flask, **1c** (850 mg, 4 mmol) was mixed with compound **D** (2.2 g, 12.8 mmol), 2.6 mL $CuSO_4 \bullet 5 H_2O$ (1 M), sodium ascorbate (520 mg, 2.7 mmol), and 12 mL of 3:1 THF/PBS buffer.[4] The reaction mixture was monitored by TLC (R_f = 0.37 (**1**), 0.41 (**2**), 100% ethyl acetate) for 6 h at room temperature (25 °C). The solvent was removed by rotary evaporation and the crude was in vacuum for 8 h. The crude oily solid was dissolved in methanol and vacuum filtered to remove the solids. The filtered methanol was removed by rotary evaporation to obtain an yellow amber oil. The yellow amber oil was purified by column chromatography from a gradient of 9:1 ethyl acetate/hexane to 100% ethyl acetate until the product was identified by TLC (R_f = 0.37 (**1**), 0.41 (**2**), 100% ethyl acetate) to obtain 40.0 %

yield of **1** (79.7% of **2**). Compounds **1** and **2** were characterized by ^{1}H NMR, FT IR and ESI-MS (Figures S15-S20).

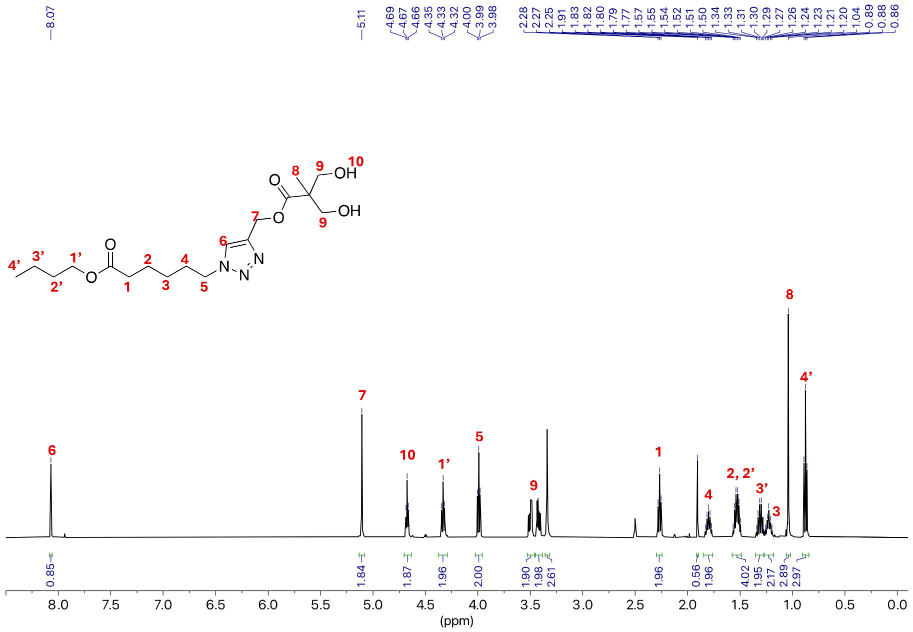

Figure S15. ^{1}H NMR (DMSO–d_6, 500 MHz, 298 K) of **1**.

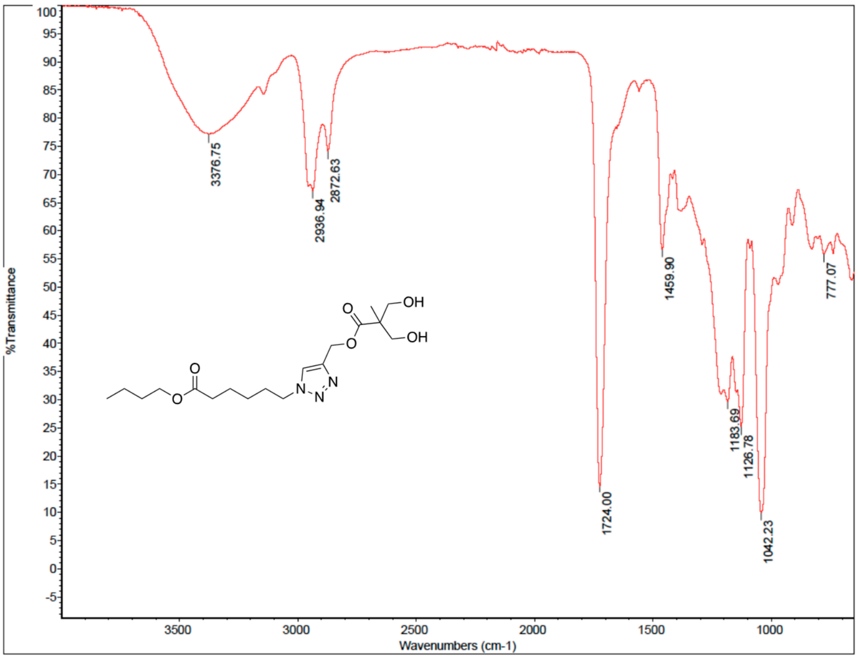

Figure S16. FTIR spectrum of final compound **1** at 298 K.

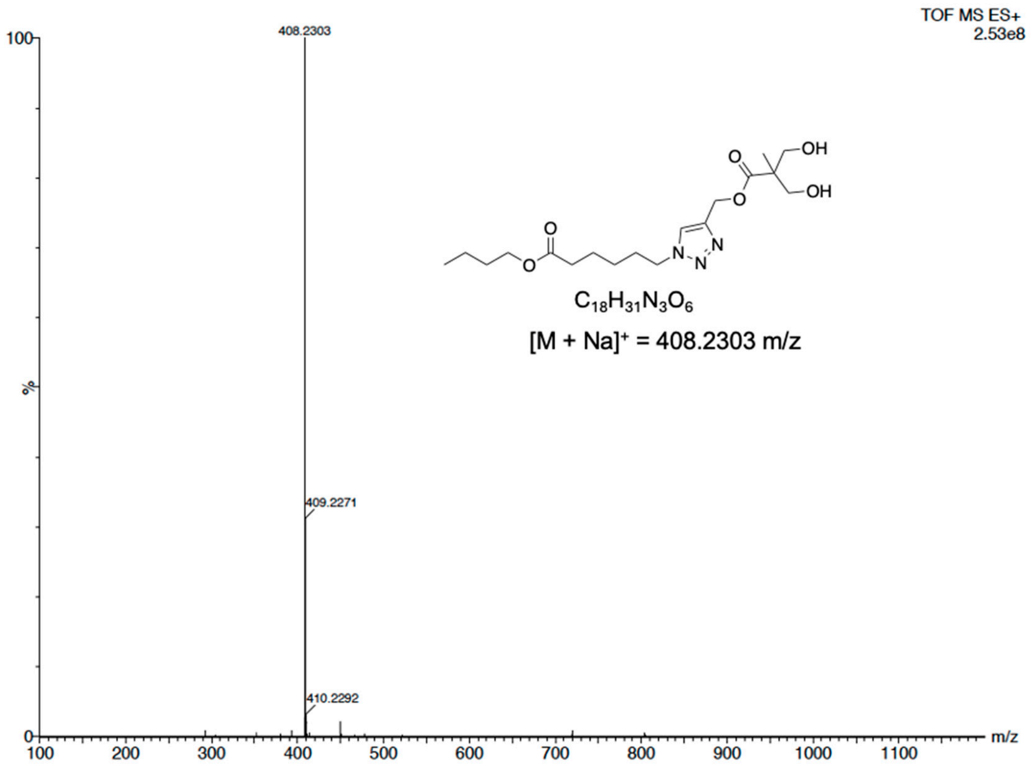

Figure S17. TOF ESI-MS analysis of final product **1**.

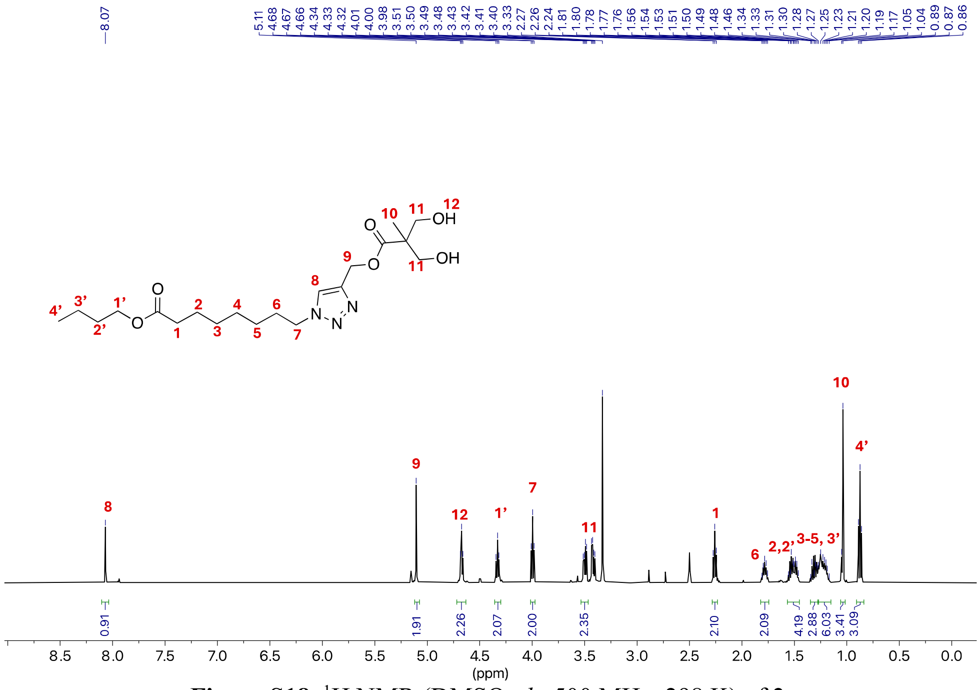

Figure S18. ^{1}H NMR (DMSO–d_6, 500 MHz, 298 K) of **2**.

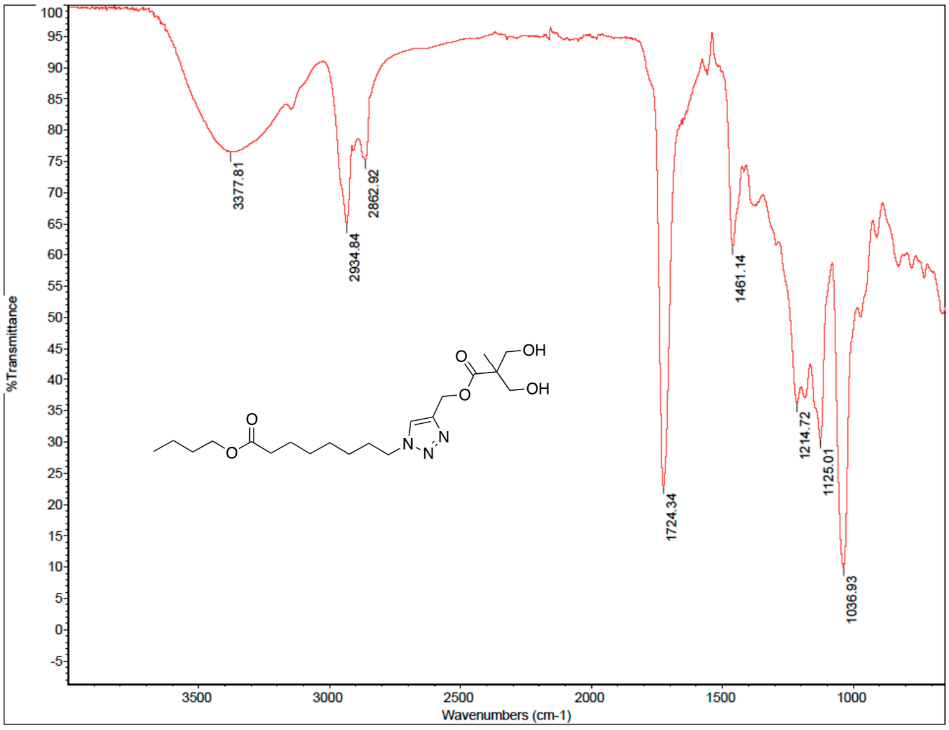

Figure S19. FTIR spectrum of final compound **2** at 298 K.

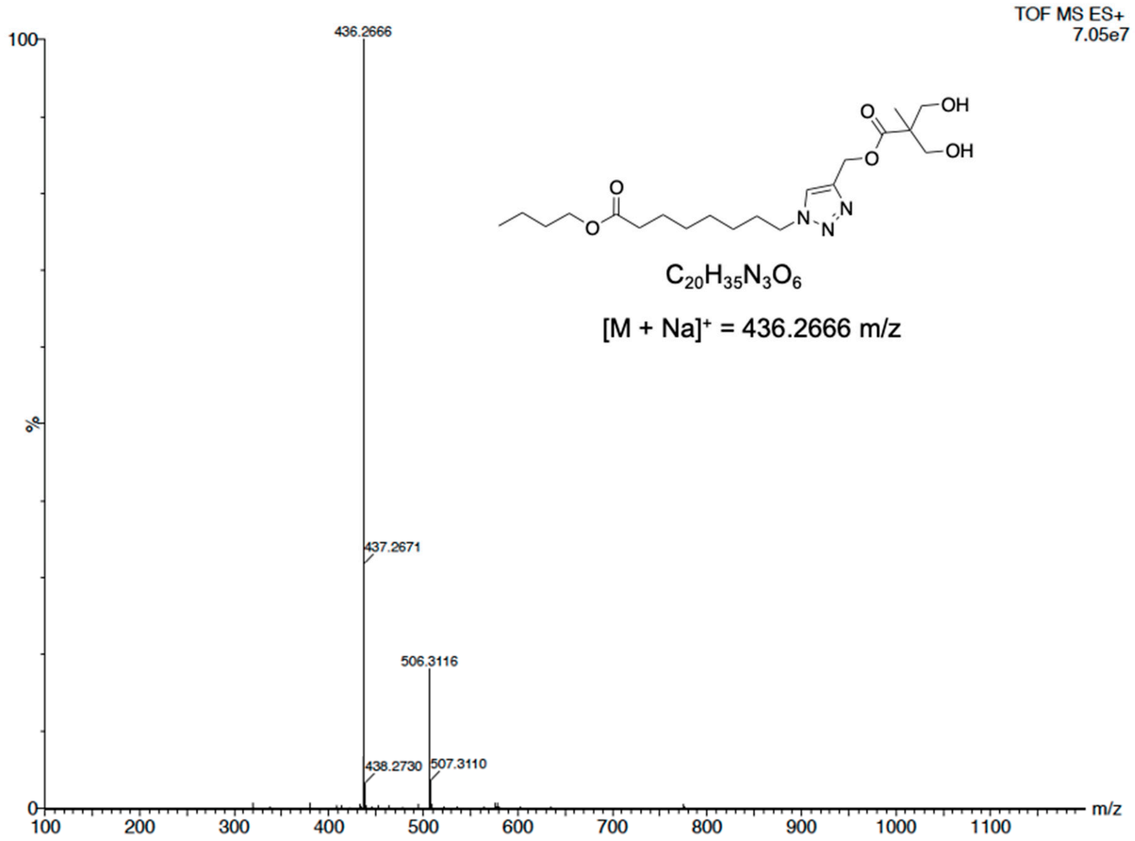

Figure S20. TOF ESI-MS analysis of final product **2**.

C. Synthesis and characterization of compound 3

The synthesis of (1-(6-(isopropylamino)-6-oxohexyl)-1H-1,2,3-triazol-4-yl)methyl 3-hydroxy-2-(hydroxymethyl)-2-methylpropanoate (**3**) was performed from 6-bromohexanoic acid (**1a**) (Figure S21).

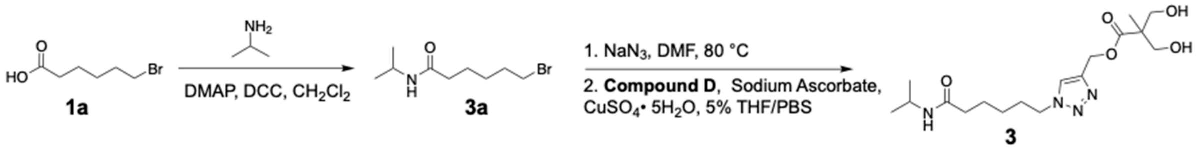

Figure S21. Synthetic scheme of **MD 3** from 6-bromohexanoic acid (**1a**).

First, intermediate **3a** was prepared by the addition of **1a** (1.5 g, 7.7 mmol), DMAP (41 mg, 3.4 mmol), isopropylamine (1.9 g, 32.1 mmol), and 25 mL of CH_2Cl_2 in a dried round bottom flask. Once the reaction mixture was partially dissolved, N, N- dicyclohexylcarbodiimide (DCM) (2.1 g, 10.2 mmol) was added for a reaction time of 12 h. Product **3a** formation was monitored by TLC (R_f = 0.68, 100% ethyl acetate) and ^{1}H NMR (Figure S22). The reaction mixture was vacuum filtered to remove the white solid dicyclohexyl urea, followed by column chromatography using a solvent gradient from 9:1 to 1:1 of hexane/ethyl acetate to obtain light yellow oil **3a** (59.1 % yield).

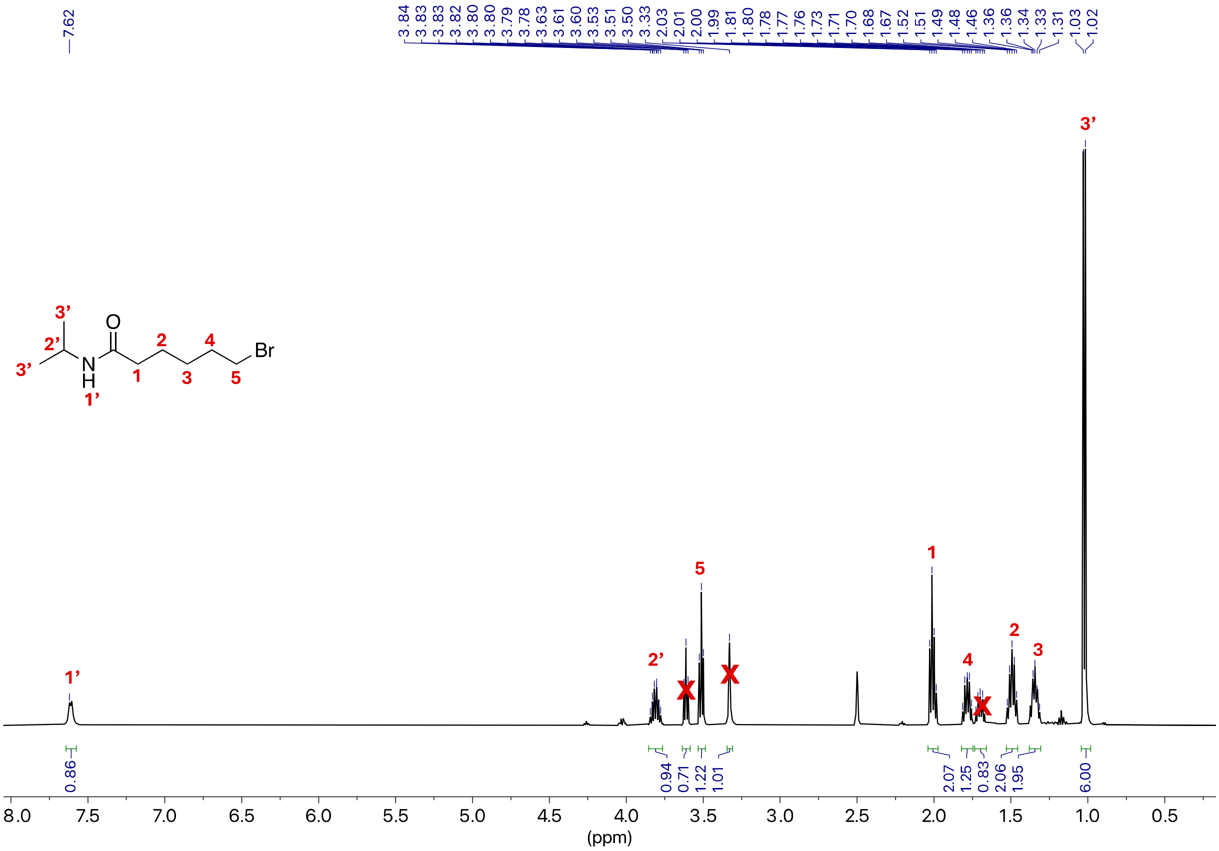

Figure S22. ^{1}H NMR (DMSO–d_6, 500 MHz, 298 K) of **3a**.

For the preparation of compound **3**, **3a** (1.1 g, 4.7 mmol) was mixed with NaN_3 (1.1 g, 17 mmol) in a round bottom flask with 15 mL of DMF for a reaction of 12 h at 80 °C. The DMF was removed by rotary evaporation, by adding 15 mL of toluene to the reaction mixture until a solid crude was formed. The solid crude was dissolved in 25 mL of methanol and the precipitated solid NaN_3 was removed by vacuum filtration. The methanol was removed by rotary evaporation and an dark amber solid identified as 6-azido-*N*-isopropylhexanamide (**3b**) (97.8 % yield, R_f = 0.72, 100% ethyl acetate). Next, to obtain product **3** by a click reaction, intermediate **3b** (0.90 g, 2.4 mmol) was mixed in the round bottom flask with compound **D** (1.2 g, 7 mmol)**,** 9.1 mL $CuSO_4 \bullet 5\ H_2O$ (1 M), sodium ascorbate (540 mg, 2.7 mmol), and 12 mL of 3:1 THF/PBS buffer. The reaction mixture was monitored by TLC (R_f = 0.27 (**3**), 9:1 ethyl acetate/methanol) for 6 h at room temperature (25 °C). The solvent was removed by rotary evaporation and the crude was in vacuum for 8 h. The crude oily solid was dissolved in methanol and vacuum filtered to remove the solids. The filtered methanol was removed by rotary evaporation to obtain an yellow amber oil. The yellow amber oil was purified by column chromatography from a gradient of 100% ethyl acetate to 9:1 ethyl acetate/methanol until the product was identified by TLC (R_f = 0.27 (**3**), 9:1 ethyl acetate/methanol) to obtain 34.0 % yield of **3**. Compound **3** was characterized by ^{1}H NMR, FT IR and ESI-MS (Figures S23-S25).

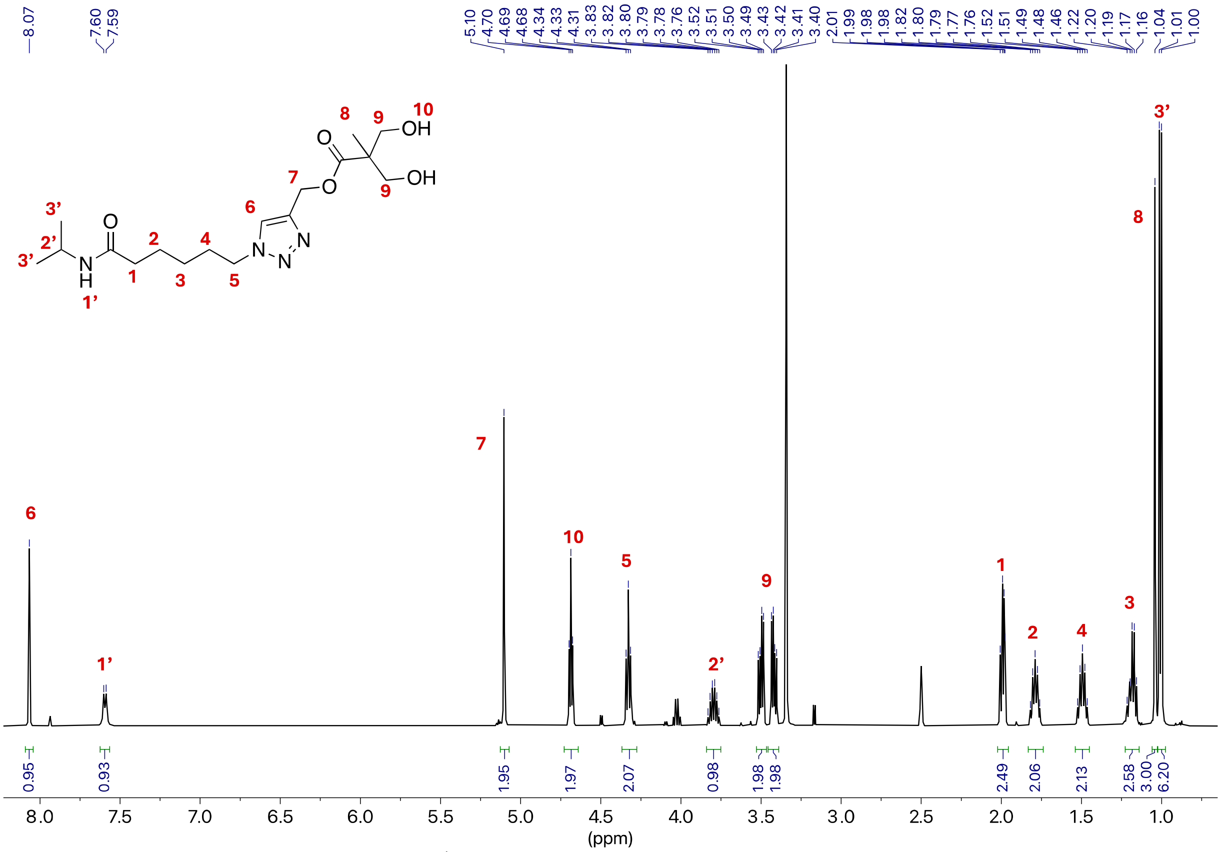

Figure S23. ^{1}H NMR (DMSO–d_6, 500 MHz, 298 K) of **3**.

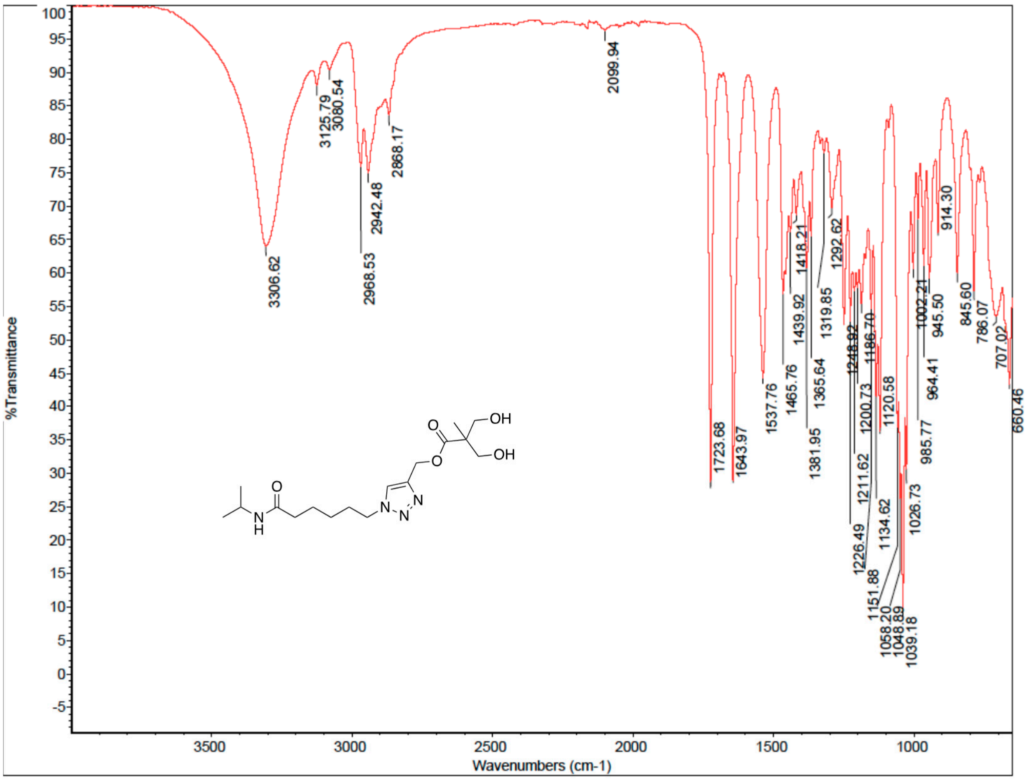

Figure S24. FTIR spectrum of final compound **3** at 298 K.

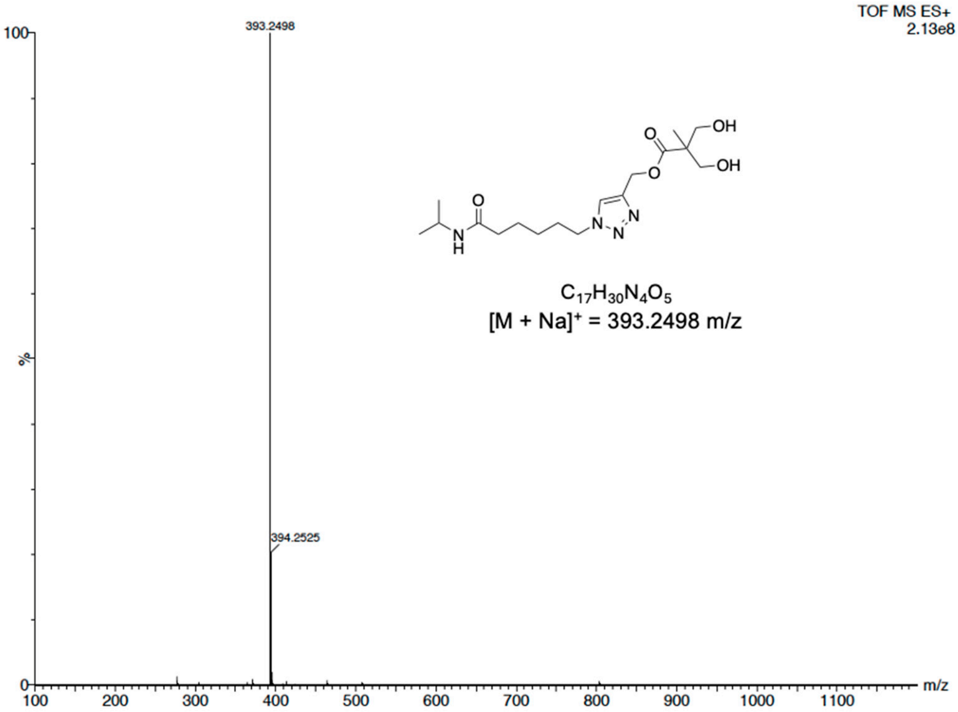

Figure S25. TOF ESI-MS analysis of final product **3**.

D. Synthesis and characterization of compound 4

Compounds **B** and **1b** described previously, were used as starting materials for the synthesis of butyl 6-((3-hydroxy-2-(hydroxymethyl)-2 methylpropanoyl)oxy) hexanoate (**4**) (Figure S26). First, **B** (1.03 g, 6 mmol) was mixed with K_2CO_3 (1.2 g, 8.8 mmol) in a round bottom flask and dissolved in 25 mL of DMF for a temperature set at 80 °C. The reaction mixture was let for 30 mins, until no CO_2 bubbles were observed in the mineral oil. Next, three portions of **1b** (0.33 g, 1.3 mmol) where then added every 20 min. Product **4a** was monitored by TLC (R_f = 0.92, 1:1 ethyl acetate/hexane) for a reaction time of 12 h.

For the workup procedure, a vacuum filtration was performed to remove the remaining solids (salts). The filtered DMF with dissolved product **4a** was mixed with 20 mL of toluene and the solution was removed by rotary evaporation until an amber oil product **4a** crude was obtained. The crude product oil was dissolved with 25 mL of CH_2Cl_2 and transferred to a separation funnel to be washed with two portions of 5 mL of distilled water. Then, the extracted CH_2Cl_2 phase was dried with $MgSO_4$ anhydrous (drying agent), followed by vacuum filtration to remove the drying agent to finally remove the filtrated CH_2Cl_2 by rotary evaporation. The resulting amber oil was purified by column chromatography using a gradient of 1:1 ethyl acetate/hexane until the product **4a** was observed by TLC (R_f = 0.92, 1:1 ethyl acetate/hexane). Purified compound **4a** was obtained with a yield of 92.8 % and characterized by ^{1}H NMR (Figure S27).

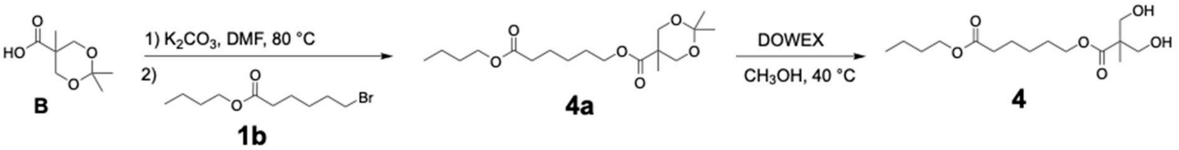

Figure S26. Synthetic scheme of **MD 4** from the combination of 2,2,5-trimethyl-1,3-dioxane-5-carboxylic acid (**B**) and butyl 6-bromohexanoate (**1b**).

Finally, compound **4** was obtained from the deprotection of intermediate **4a** with DOWEX 50WX2 resin. In a round bottom flask, compound **4a** (1.3 g, 3.8 mmol) was dissolved in 20 mL of methanol. Followed by the addition of 1.3 g of DOWEX 50WX2 resin to let the reaction mixture for 12 h at 40 °C. The reaction was monitored by TLC (compound **4**, R_f = 0.71, 100 % ethyl acetate), once the product was formed the solid DOWEX 50WX2 resin was vacuum filtered and the methanol was removed by rotary evaporation to obtain an amber color oil product **4** (64.8 % yield) observed by ^{1}H NMR, FT-IR and ESI-MS (Figure S28-30).

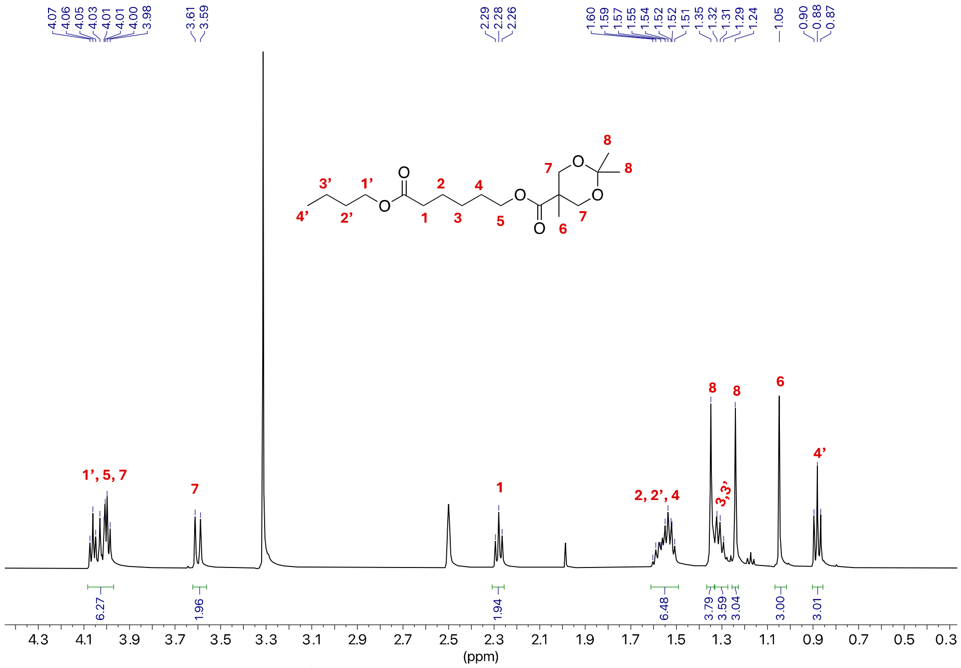

Figure S27. ^{1}H NMR (DMSO–d_6, 500 MHz, 298 K) of **4a**.

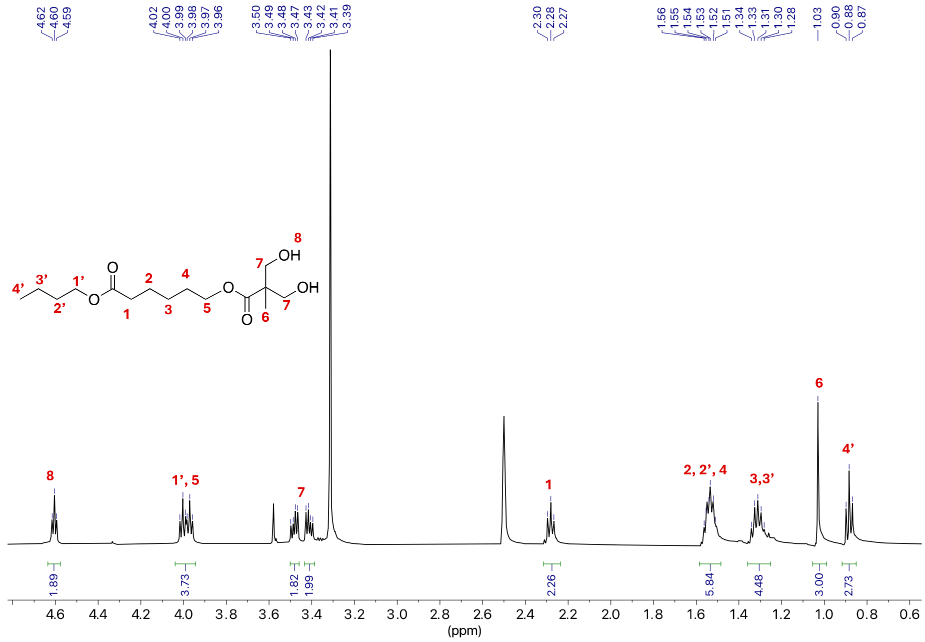

Figure S28. ^{1}H NMR (DMSO–d_6, 500 MHz, 298 K) of **4**.

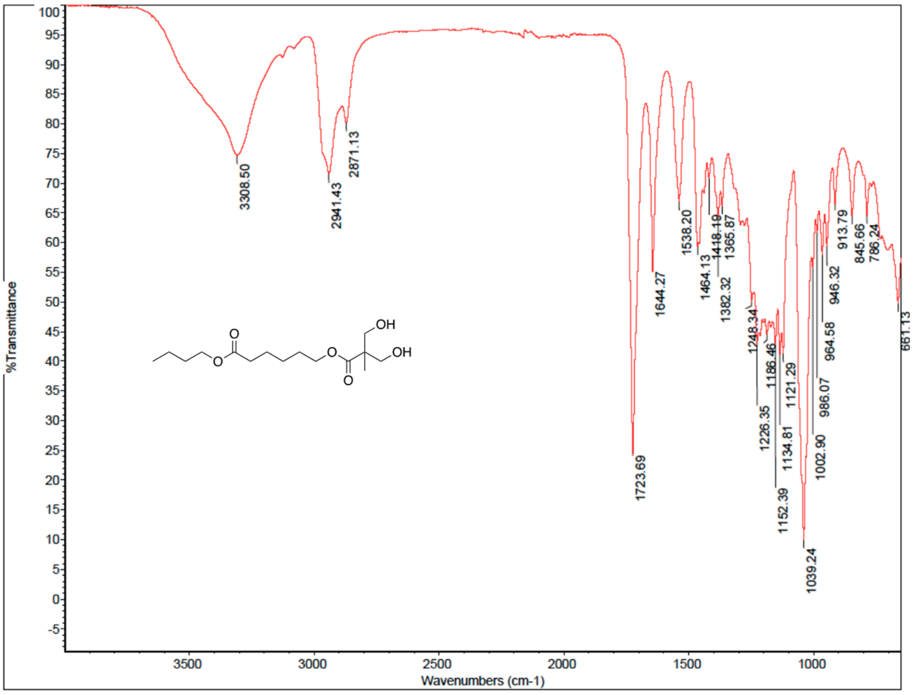

Figure S29. FTIR spectrum of final product **4** at 298 K.

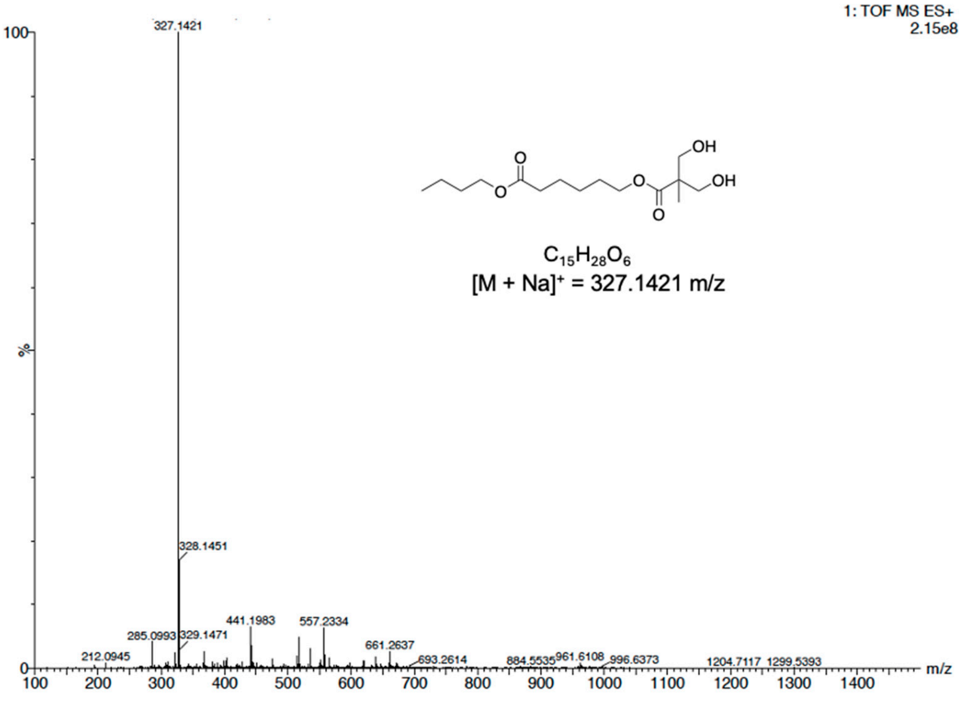

Figure S30. TOF ESI-MS analysis of final product **4**.

E. Synthesis and characterization of compound 5

For the synthesis and characterization of hexane-1,6-diyl bis(3-hydroxy-2-(hydroxymethyl)-2-methylpropanoate) (**5**), compound 5 was prepared using a similar procedure used for compound **4** (Figure S31). First, 1,6-dibromohexane (**5a**) was prepared by a procedure from Pavia, by the addition of 1,6-hexanediol (4.1 g, 34.7 mmol), NaBr (17 g, 155 mmol) and 17 mL of distilled water in a round bottom flask. The mixture was cooled in an ice bath for the addition of 14 mL of H_2SO_4 (17.8 M). The reaction mixture was under reflux at 80 °C for 2 h, followed by the observation of two phases in the round bottom flask. In a separation funnel, the lower aqueous phase was removed, followed by washing the organic phase (**5a**) with 14 mL of H_2SO_4 (9 M). Next, the aqueous H_2SO_4 phase was removed, and the organic phase was washed with 14 mL of distilled water. The third and last wash was performed with 14 mL of saturated Na_2CO_3 solution. The lower organic phase (**5a**) was removed and dried with $MgSO_4$ anhydrous. After removal of the $MgSO_4$ anhydrous by vacuum filtration, compound **5a** (33 % yield) and starting material (1,6-hexanediol) were monitored by TLC (R_f = 0.89, 100% ethyl acetate), ^{1}H NMR and FTIR (Figure S32-S35).

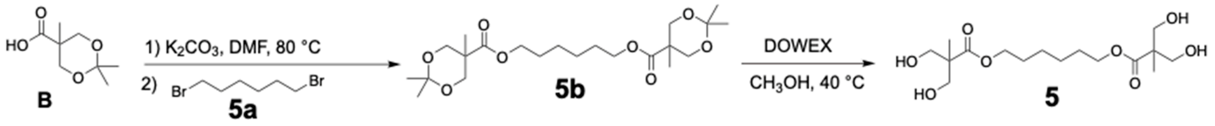

Figure S31. Synthetic scheme of **MD 5** from thre combination of 2,2,5-trimethyl-1,3-dioxane-5-carboxylic acid (**B**) and 1,6-dibromohexane (**5a**).

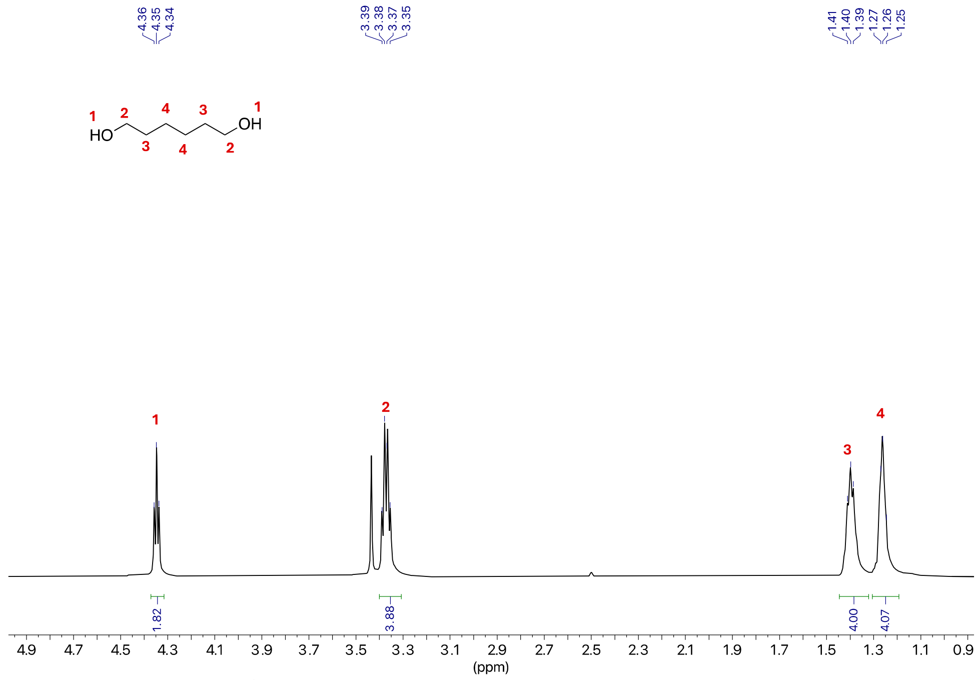

Figure S32. ^{1}H NMR (DMSO–d_6, 500 MHz, 298 K) of **1,6-hexanediol**.

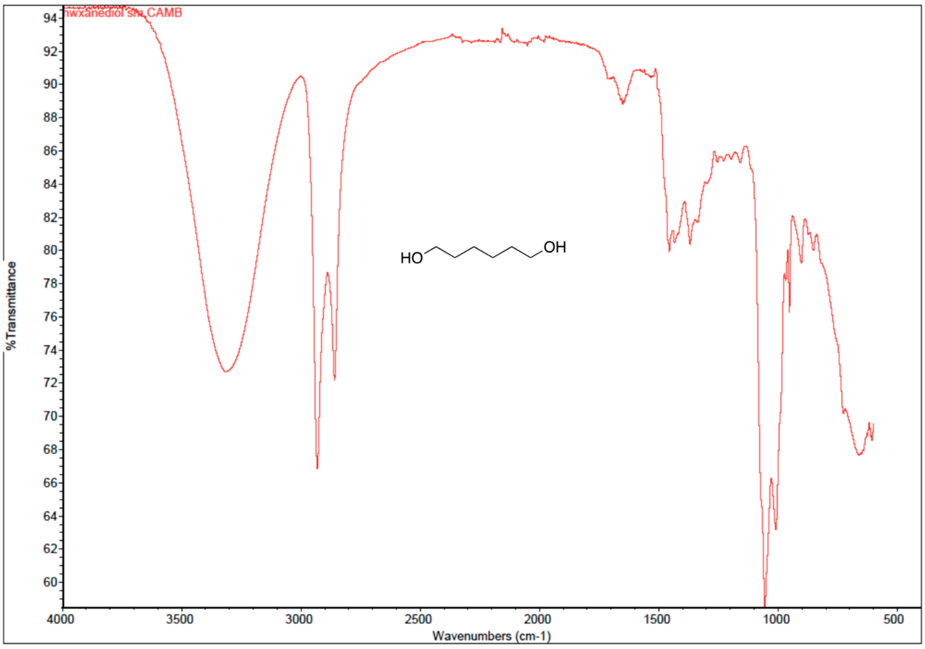

Figure S33. FTIR spectrum of starting material **1,6-hexanediol** at 298 K.

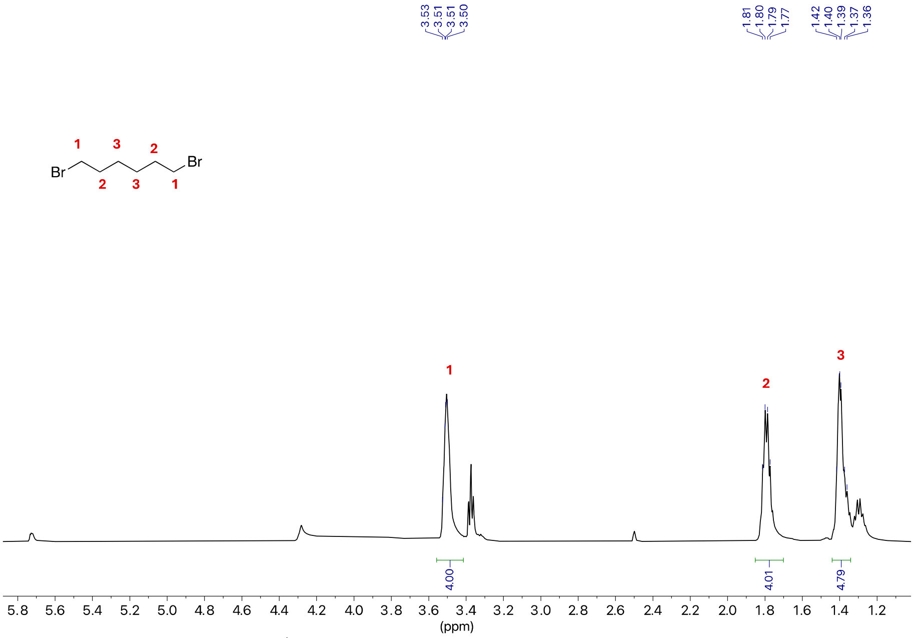

Figure S34. ^{1}H NMR (DMSO–d_6, 500 MHz, 298 K) of **5a**.

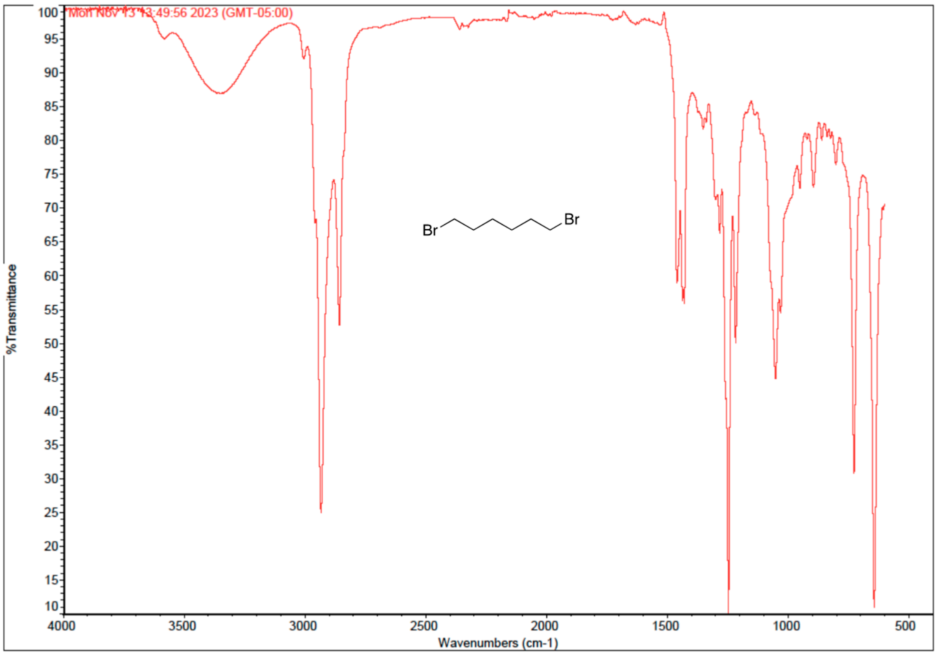

Figure S35. FTIR spectrum of intermediate **5a** at 298 K.

For the synthesis and characterization of hexane-1,6-diyl bis(2,2,5-trimethyl-1,3-dioxane-5-carboxylate) (**5b**), compound **B** (1.4 g, 27.3 mmol) was mixed with K_2CO_3 (7.5 g, 54.3 mmol) in a round bottom flask and dissolved in 25 mL of DMF for a temperature set at 80 °C. The reaction mixture was let for 30 mins, until no CO_2 bubbles were observed in the mineral oil. Next, two portions of **5a** (0.7 g, 2.9 mmol) where then added every 20 min. Product **5b** was monitored by TLC (R_f = 0.86, 100% ethyl acetate) for a reaction time of 12 h. For the workup procedure, a vacuum filtration was performed to remove the remaining solids (salts). The filtered DMF with dissolved product **5b** was mixed with 20 mL of toluene and the solution was removed by rotary evaporation until an amber oil product **5b** crude was obtained. The crude product oil was dissolved with 25 mL of CH_2Cl_2 and transferred to a separation funnel to be washed with two portions of 5 mL of distilled water. Then, the extracted CH_2Cl_2 phase was dried with $MgSO_4$ anhydrous (drying agent), followed by vacuum filtration to remove the drying agent to finally remove the filtrated CH_2Cl_2 by rotary evaporation. The resulting light-yellow oil was purified by column chromatography using a gradient of 1:1 ethyl acetate/hexane until the product **5b** was observed by TLC (R_f = 0.86, 100% ethyl acetate). Purified compound **5b** was obtained with a yield of 30.0 % and characterized by ^{1}H NMR and FTIR (Figure S36-S37).

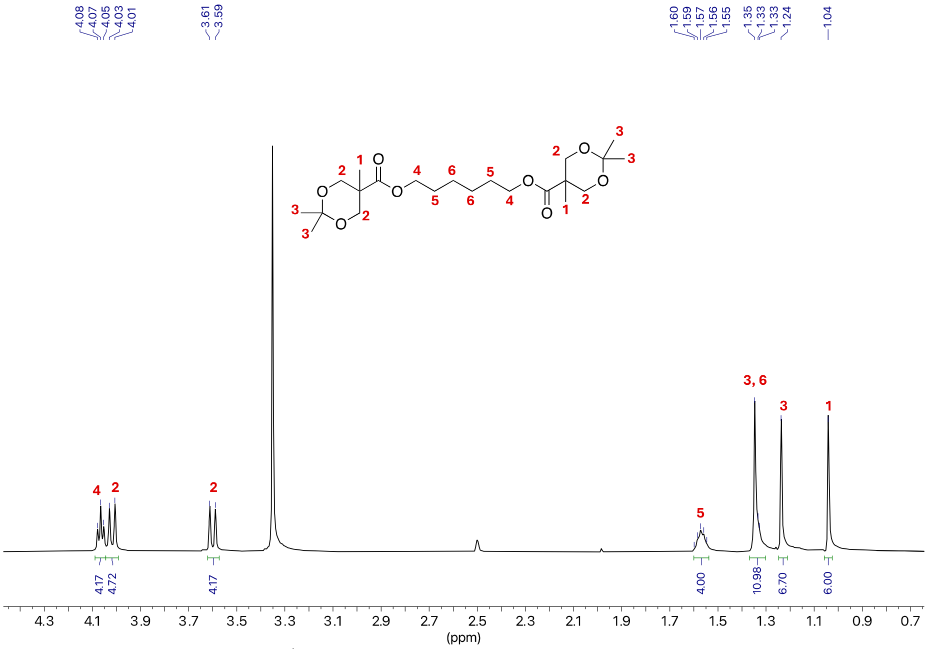

Figure S36. ^{1}H NMR (DMSO–d_6, 500 MHz, 298 K) of **5b**.

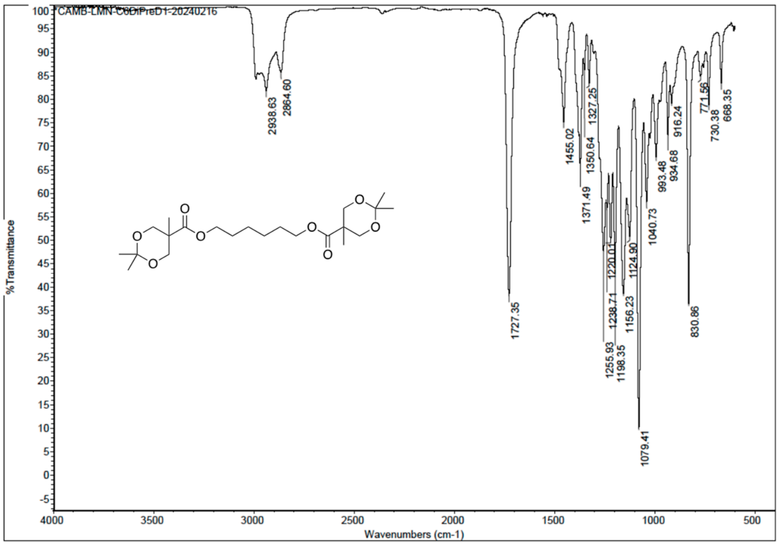

Figure S37. FTIR spectrum of intermediate **5b** at 298 K.

Finally, compound **5** was obtained from the deprotection of intermediate **5b** with DOWEX 50WX2 resin. In a round bottom flask, compound **5b** (0.7 g, 1.7 mmol) was dissolved in 20 mL of methanol. Followed by the addition of 1.0 g of DOWEX 50WX2 resin to let the reaction mixture for 12 h at 40 °C. The reaction was monitored by TLC (Compound **5**, R_f = 0.31, 100 % ethyl acetate), once the product was formed the solid DOWEX 50WX2 resin was vacuum filtered and the methanol was removed by rotary evaporation to obtain an amber color oil product **5** (85.4 % yield) observed by ^{1}H NMR, FT-IR and ESI-MS (Figure S28-30).

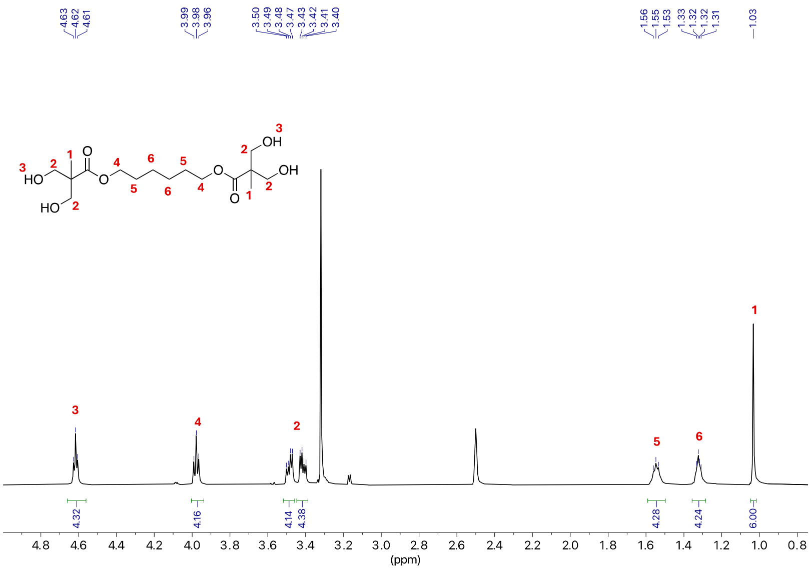

Figure S38. ^{1}H NMR (DMSO–d_6, 500 MHz, 298 K) of **5**.

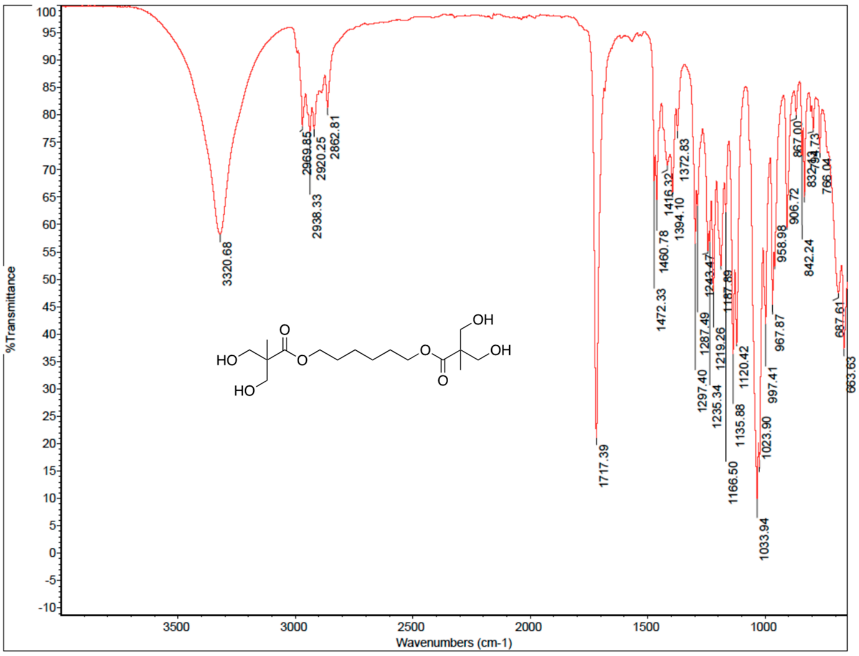

Figure S39. FTIR spectrum of final product **5** at 298 K.

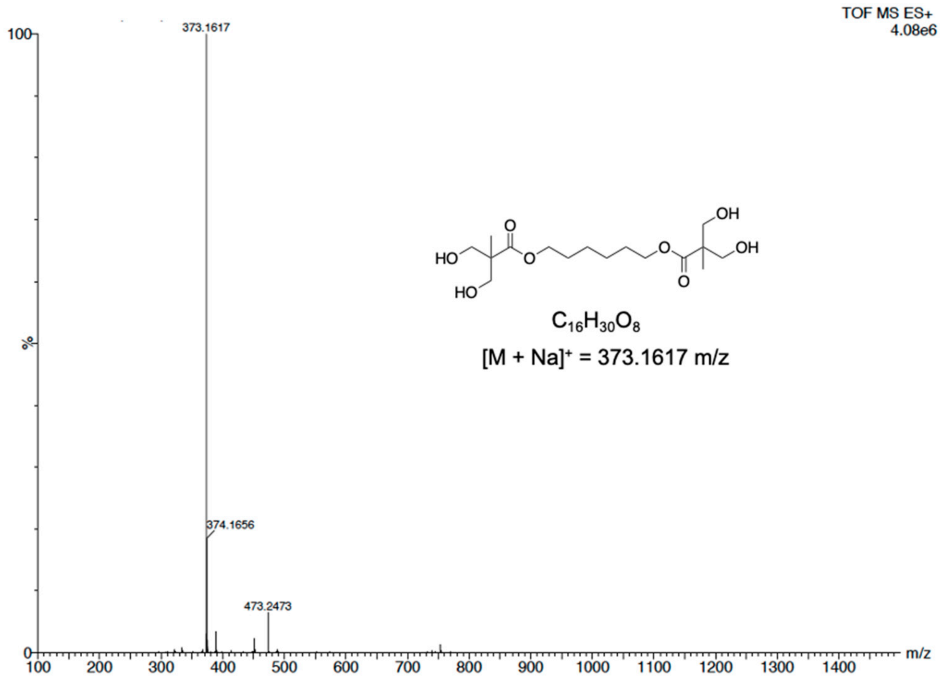

Figure S40. TOF ESI-MS analysis of final product **5**.

IV. MDP formation protocol

All the **1-5 MDs** (Figure S1) were prepared using a dendron concentration of 15 mM in PBS. For example, for **MDP 2**, 6.2 mg of **D2** were added to a 7 mL glass vial followed by the addition of 1.00 mL of PBS. The sample vial was placed in a commercially available 2 L ultrasonic bath from VEVOR (40 kHz ultrasonic frequency, 60 W ultrasonic Power) for 1 minute at 25 °C (Figure S41).

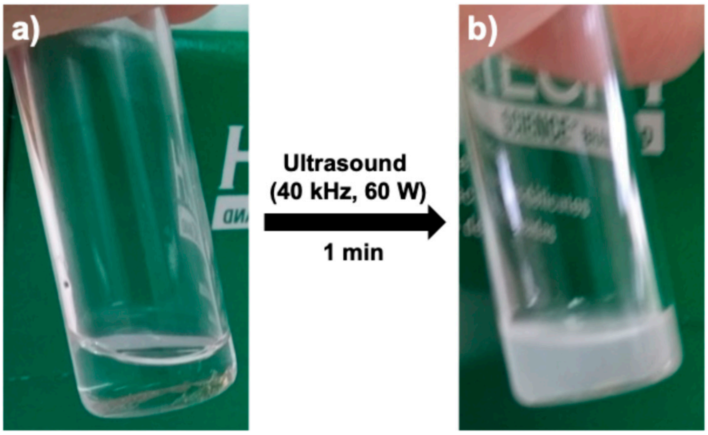

Figure S41. Representation for sample preparation of MDP 2 using ultrasound for 1 min.

Once the added dendron derivative (oil or solid) was not observed in the PBS, the resulting colloidal suspension of MDP was ready to perform the following studies: SEM, DLS, CLSM and cell viability studies with MTS assay. The appearance of the formed colloidal suspension depended on the type of **MDs 1-5** used in which for MDs **2**, **3** and **5** were more intense than MDs **1** and **4**.

V. Scanning Electron Microscopy (SEM) characterization

For the SEM experiments, MDP samples were prepared by the procedure discussed in **section IV**. Once the MDP was prepared, 10 µL of MDP was added dropwise in a Holey carbon supported copper grid (200 mesh, 100 nm). The grid samples were dried in a fume hood at room temperature (25 °C) for 12 hrs. After the sample drop over the grid was dried, the sample grid was placed in a Pelco SC-7 Auto Sputter Coater for gold coating for 30 min.

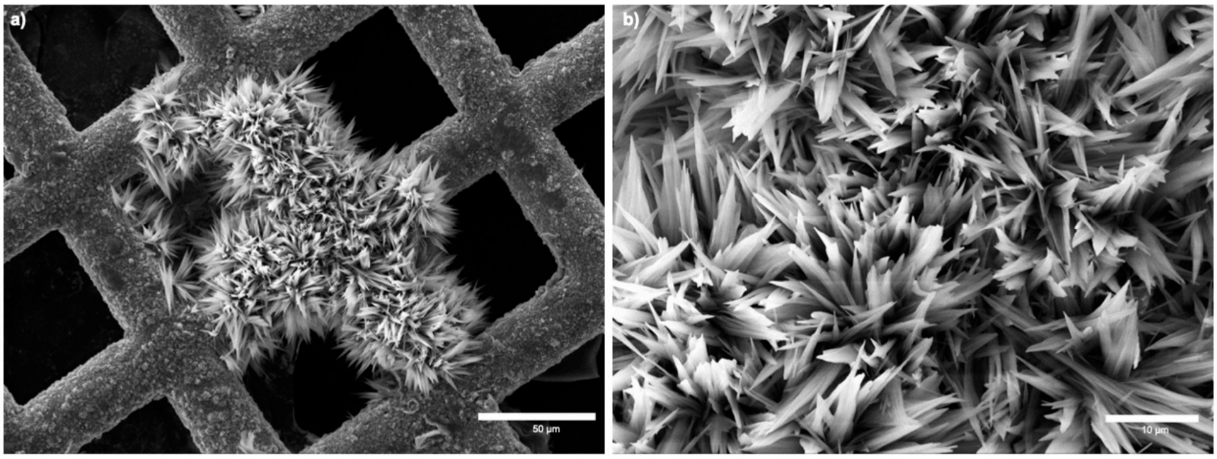

Figure S42. SEM images of **MDP 1** (15 mM of **MD 1**, SED 20.0 kV, WD 11 mm) with different magnifications at x500 (a) and x2,000 (b).

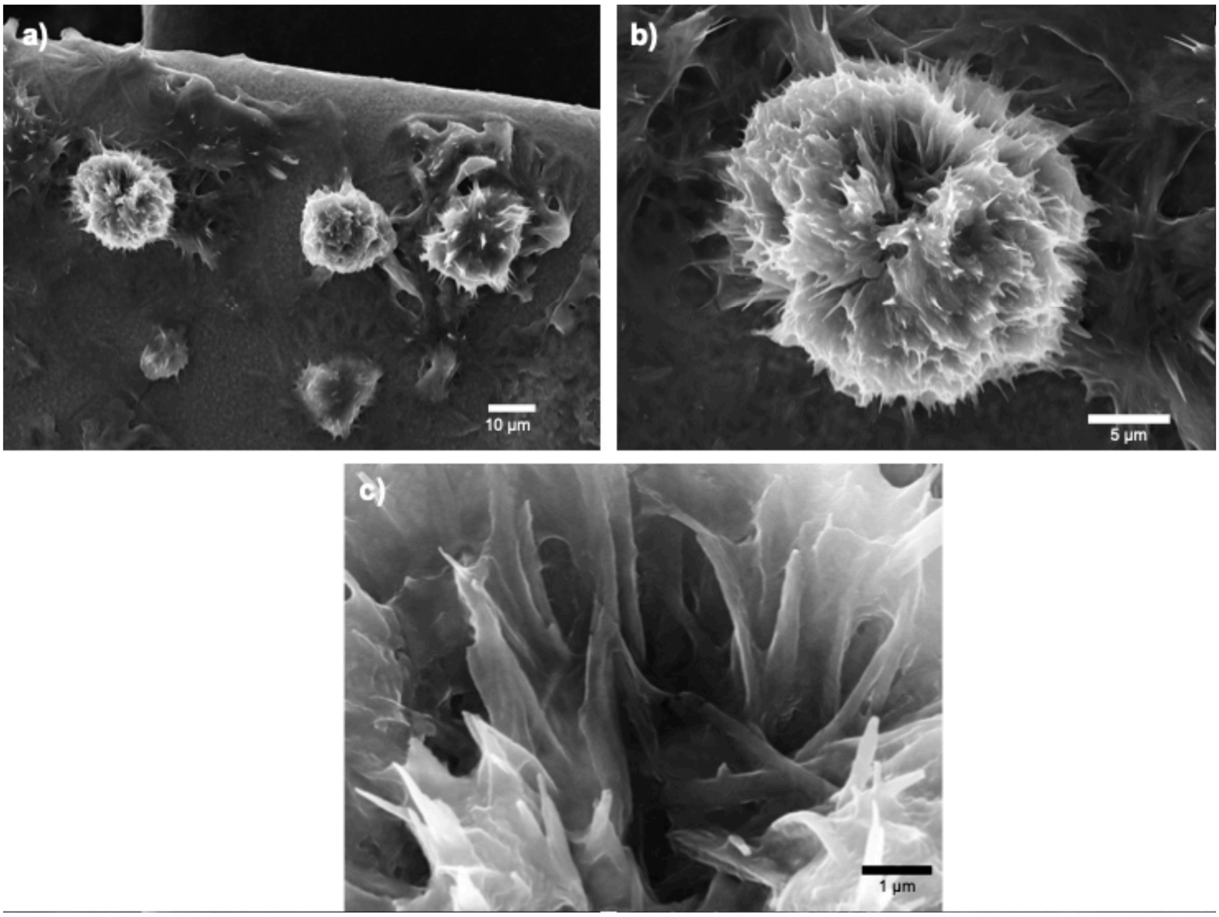

Figure S43. SEM images of **MDP 2** (15 mM of **MD 2**, SED 20.0 kV, WD 11 mm) with different magnifications at x1,000 (a), x3,500 (b), and x15,000 (c).

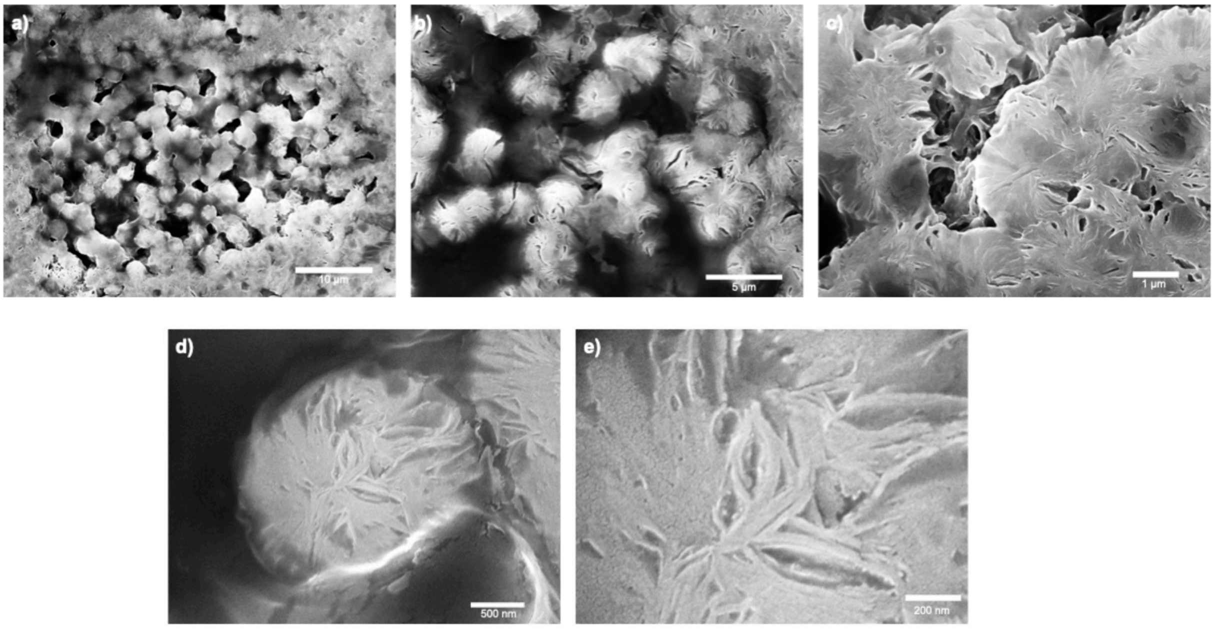

Figure S44. SEM images of **MDP 3** (15 mM of **MD 3**, SED 20.0 kV, WD 11 mm) with different magnifications at x2,500 (a), x5,000 (b), x15,000 (c), x35,000 (d), and x90,000 (e).

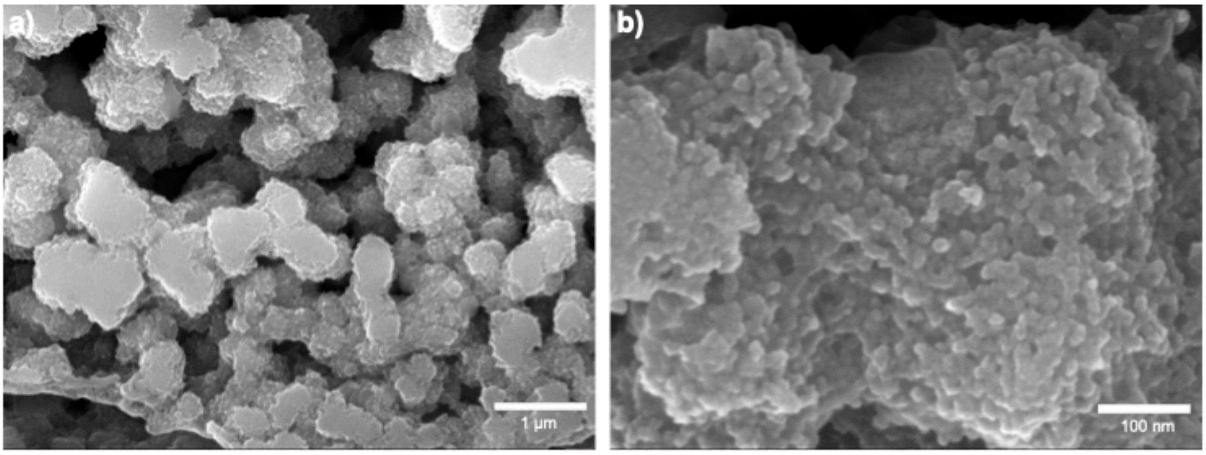

Figure S45. SEM images of **MDP 4** (15 mM of **MD 4**, SED 15.0 kV, WD 11 mm) with different magnifications at x20,000 (a) and x100,000 (b).

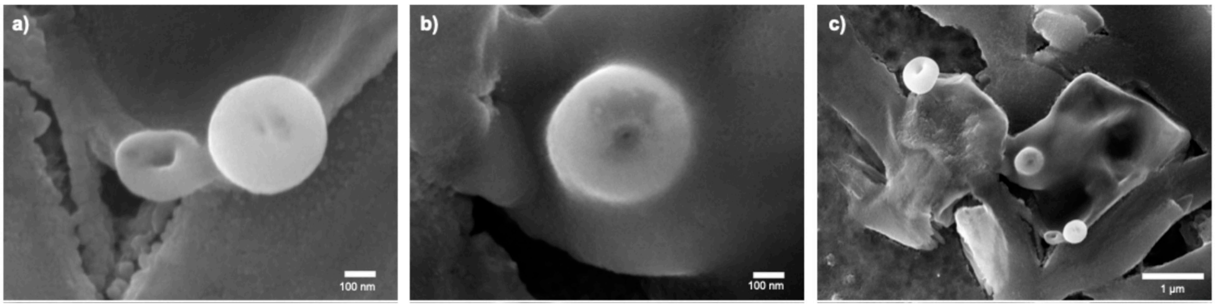

Figure S46. SEM images of **MDP 5** (15 mM of **MD 5**, SED 10.0 kV, WD 11 mm) at different magnifications of x100,000 (a), x100,000 (b), and x20,000 (c).

VI. Cell culture studies

The selected cell lines Neuro-2a neuroblastoma cells (N2a) and bronchial epithelial (NL-20) were incubated in a ST-180 CO_2 humidified incubator from Benchmark Scientific at 37.1 °C and 5 % CO_2. Both cell lines were seeded at a density of 1×10^5 cells per well in 96-well plates in a final volume of 200 µL per well. N2a cells were cultured in Eagle's Minimum Essential Medium (EMEM; ATCC 30-2003) supplemented with 10% fetal bovine serum (FBS; Gibco A5256701). NL-20 cells (ATCC CRL-2503) were cultured in Airway Epithelial Cell Basal Medium (ATCC PCS-300-030) supplemented with the Bronchial Epithelial Cell Growth Kit (ATCC PCS-300-040).

For cryopreservation, cells were suspended in freezing medium containing 5% dimethyl sulfoxide (DMSO) and stored at -80 °C in an ultra-low temperature freezer (Haier, Model: DW-86L578J). Cells were harvested using a swing-bucket centrifuge (Benchmark, Model: Hermle Z327K) at 1200 rpm for 5 min. Cell morphology and confluency were routinely monitored using a 20× brightfield microscope (Nikon, Model: Eclipse Ts2).

VII. MTS cell viability studies

Cell proliferation experiments were performed using a colorimetric MTS assay kit (from BioVision, purchased in Abcam) according to the manufacturer's instructions. To evaluate the

effects of each MDP on different cell types, cancerous N2a and non-cancerous NL-20 cell lines were incubated for 24 h and subsequently assessed for viability using the MTS assay.

For each MDP, cell viability was evaluated at the following concentrations: 0 mM (control), 0.4 mM, 2 mM, and 4 mM. Untreated cells served as the negative control (representing 100 % viability), whereas cells treated with 3 % H_2O_2 were used as the positive control for cytotoxicity. Wells containing culture medium and MTS reagent without cells were included as blanks to correct for background absorbance. After treatment, 20 µL of MTS reagent was added to each well, and the plates were incubated for an additional 2 h at 37 °C. Absorbance was measured at 490 nm using a SpectraMax M3 Multi-Mode Microplate Reader (Molecular Devices).

Cell viability was calculated from absorbance values following background subtraction and normalization to untreated control cells. In some cases, background-corrected absorbance values at high MDP concentrations approached the instrumental noise level, resulting in small negative values upon normalization. Because negative cell viability has no physical or biological meaning, values below 0% were set to 0% viability for data presentation. This conservative truncation does not affect the qualitative trends or comparative analysis of cellular responses across dendron structures and concentrations.

VIII. Dynamic Light Scattering (DLS)

Dynamic light scattering (DLS) was employed to determine the hydrodynamic diameters of the modular dendron particles (MDPs) in aqueous media. Measurements were carried out at 25.0 ± 0.1 °C using a Zetasizer Nano ZS (ZEN3600, Malvern Instruments Ltd.). Samples were prepared by sonication of 15 mM dendron solutions in PBS (pH 7.4) for 1 min prior to analysis. Hydrodynamic diameters were obtained from cumulants analysis and are reported as Z-average values. Each sample was measured in triplicate ($n = 3$), with each measurement consisting of an average of 11 runs after an equilibration time of 60 s.

The reported Z-average values and corresponding polydispersity indices (PDI) are expressed as mean ± standard deviation and are summarized in Table S1. PDI values were extracted directly from the instrument software and provide a qualitative assessment of the breadth of the particle size distributions. Minor variations in temperature across measurements (< ±0.1 °C) were considered negligible and did not affect the reported size trends.

Table S1. Z-average hydrodynamic diameters and polydispersity index (PDI) values of modular dendron particles (MDPs) obtained by dynamic light scattering (DLS). Measurements were performed at 25.0 ± 0.1 °C on samples prepared by sonication of 15 mM dendron solutions in PBS (pH 7.4) for 1 min. Z-average values were obtained from cumulants analysis and are reported as mean ± standard deviation from three independent measurements ($n = 3$).

MDP	Z-Average (nm)	PDI
**1**	532 ± 74	0.48 ± 0.09
**2**	3490 ± 229	0.17 ± 0.03
**3**	229 ± 4	0.32 ± 0.03
**4**	652 ± 53	0.16 ± 0.11
**5**	359 ± 2	0.51 ± 0.03

References

(1) Ihre, H.; Hult, A.; Fréchet, J. M. J.; Gitsov, I. Double-Stage Convergent Approach for the Synthesis of Functionalized Dendritic Aliphatic Polyesters Based on 2,2-Bis(Hydroxymethyl)Propionic Acid. *Macromolecules* **1998**, *31* (13), 4061–4068. https://doi.org/10.1021/ma9718762

(2) Malkoch, M.; Malmström, E.; Hult, A. Rapid and Efficient Synthesis of Aliphatic Ester Dendrons and Dendrimers. *Macromolecules* **2002**, *35* (22), 8307–8314. https://doi.org/10.1021/ma0205360

(3) Turhanen, P. A.; Leppänen, J.; Vepsäläinen, J. J. Green and Efficient Esterification Method Using Dried Dowex H^+ /NaI Approach. *ACS Omega* **2019**, *4* (5), 8974–8984. https://doi.org/10.1021/acsomega.9b00790

(4) Negrón, L. M.; Meléndez-Contés, Y.; Rivera, J. M. Patchy Supramolecules as Versatile Tools To Probe Hydrophobicity in Nanoglobular Systems. *J. Am. Chem. Soc.* **2013**, *135* (10), 3815–3817. https://doi.org/10.1021/ja401373h
